# Grass Carp Prolactin Gene: Structural Characterization and Signal Transduction for PACAP-induced Prolactin Promoter Activity

**DOI:** 10.1038/s41598-018-23092-0

**Published:** 2018-03-15

**Authors:** Chengyuan Lin, Jin Bai, Mulan He, Anderson O. L. Wong

**Affiliations:** 1 School of Biological Sciences, University of Hong Kong, Hong Kong HKSAR, China; 2 Clinical Division, School of Chinese Medicine, Hong Kong Baptist University, Hong Kong HKSAR, China; 3 YMU-HKBU Joint Laboratory of Traditional Natural Medicine, Yunnan Minzu University, Kunming, China

## Abstract

In this study, structural analysis of grass carp prolactin (PRL) gene was performed and the signaling mechanisms for pituitary adenylate cyclase-activating peptide (PACAP) regulation of PRL promoter activity were investigated. In αT3-1 cells, PRL promoter activity could be induced by oPACAP_38_ which was blocked by PACAP antagonist but not the VIP antagonist. The stimulatory effect of oPACAP_38_ was mimicked by activation of AC/cAMP and voltage-sensitive Ca^2+^ channel (VSCC) signaling, or induction of Ca^2+^ entry. In parallel, PACAP-induced PRL promoter activity was negated or inhibited by suppressing cAMP production, inhibiting PKA activity, removal of extracellular Ca^2+^, VSCC blockade, calmodulin (CaM) antagonism, and inactivation of CaM kinase II. Similar sensitivity to L-type VSCC, CaM and CaM kinase II inhibition were also observed by substituting cAMP analog for oPACAP_38_ as the stimulant for PRL promoter activity. Moreover, PACAP-induced PRL promoter activity was also blocked by inhibition of PLC signaling, attenuation of [Ca^2+^]i immobilization via IP3 receptors, and blockade of PI3K/P_70_^S6K^ pathway. The PACAP-induced PRL promoter activation may involve transactivation of the transcription factor CREB. These results suggest that PACAP can stimulate PRL promoter activation by PAC1 mediated functional coupling of the Ca^2+^/CaM/CaM kinase II cascades with the AC/cAMP/PKA pathway. Apparently, other signaling pathways, including PLC/IP3 and PI3K/P_70_^S6K^ cascades, may also be involved in PACAP induction of PRL gene transcription.

## Introduction

Prolactin (PRL) is a pituitary hormone with diverse function. Phylogenetic analysis of gene sequences has revealed that PRL, growth hormone (GH), and somatolactin are evolved from the same ancestral gene by gene duplication^1^. In mammals, transcription of PRL gene is regulated by multiple factors. Dopamine is well-documented to exert inhibitory effect on PRL promoter activity via dopamine D2 receptor activation^2^. In contrast, thyrotropin-releasing hormone (TRH) is considered to be a PRL-releasing factor, which can stimulate PRL promoter via pit-1 transactivation^3^. Furthermore, sex steroids, especially estrogen, have been proposed to serve as a potent stimulator for PRL gene transcription (e.g., in human), presumably by functional interactions of estrogen receptor (ER) and activator protein 1 (AP1) transcription factors on the respective cis-acting elements in the PRL promoter^4,5^. Unlike mammalian studies, no much information is available on PRL promoter regulation in non-mammalian vertebrates. In goldfish, dopamine inhibition of PRL promoter activity can be observed^6^. However, PRL transcript expression and PRL promoter activity were both suppressed by TRH treatment in the same study. This discrepancy of TRH actions between the fish model and mammals suggests that the neuroendocrine regulation of PRL gene expression might have been altered/modified during the course of vertebrate evolution.

PACAP and vasoactive intestinal peptide (VIP) are members of the glucagon/secretin peptide family and have been reported to play roles in PRL regulation^7^. In mammals, the stimulatory effects of VIP on PRL secretion^8^ and gene expression^9^ have been well-documented, and multiple sites of actions, namely actions within the central nervous system (e.g., via modulation of DA neuronal activity)^10^, direct stimulation of lactotrophs at the pituitary level^11^, or by autocrine/paracrine mechanisms within the pituitary^12^ have been reported. In contrast to VIP, the biological actions of PACAP on PRL secretion have been controversial, as stimulatory^9,13^, inhibitory^14^, and no effect^15,16^ have been reported. In the case of stimulatory effects, e.g., in GH3 cells, PACAP can up-regulate PRL promoter activity via activation of cyclic adenosine 3′,5′-monophosphate (cAMP)-dependent signaling mechanism^17^. In GH4C1 cells, the stimulatory effects of PACAP and VIP on PRL gene expression are mediated through vasoactive intestinal peptide receptor 2 (VPAC1I) functionally coupled with Ras and Rap small GTPase^18^. VPAC receptors, formerly known as the VIP receptors, are known to bind PACAP and VIP with similar affinity, and its pharmacological properties is quite distinct from the PAC1 receptors for PACAP, which is specific for PACAP with little/very low affinity for VIP^19^.

In carp model, PACAP nerve fibers can be identified in anterior pituitary overlapping with the distribution of lactotrophs^20^. Furthermore, PAC1 receptors could be detected in grass carp lactotrophs. PRL secretion and PRL mRNA expression were up-regulated by PACAP but not VIP treatment via functional coupling of Ca^2+^/calmodulin (CaM)- and c-Jun N-terminal kinase (JNK)-dependent cascades with the cAMP/PKA pathways^21^. These findings suggest that PACAP but not VIP can serve as a stimulator for PRL synthesis in the carp species and this stimulatory action is mediated through activation of PAC1 but not VPAC receptors at the pituitary level. To further elucidate the mechanisms for PACAP regulation of grass carp PRL gene expression, the effect of PACAP treatment on PRL promoter activity and the post-receptor signaling cascades involved were examined in αT3-1 cells. The full gene of grass carp PRL was cloned by genome walking and the transcription initiation site was defined by primer extension. The 5′ promoter of grass carp PRL gene was then subcloned into a luciferase-expressing reporter for promoter studies in αT3-1 cells. PACAP responsive sequence of grass carp PRL promoter was mapped by 5′ deletion analysis. The functional involvement of the cAMP/PKA, Ca^2+^/CaM/CaM Kinase-, mitogen-activated protein kinase (MAPK)- and phosphoinositide 3-kinase (PI3K)-dependent cascades were tested with the pharmacological agents that could perturb the respective pathways. To shed light on the transcription factors mediating the genomic action of PACAP in terms of PRL gene transcription, the effect of over-expression of cAMP response element binding protein (CREB) on basal PRL promoter activity and siRNA/domain negative (DN) mutant of CREB on PACAP-induced PRL promoter activity were also investigated in αT3-1 cells.

## Results

### Molecular Cloning of Grass Carp Prolactin Gene

**Figure 1:**
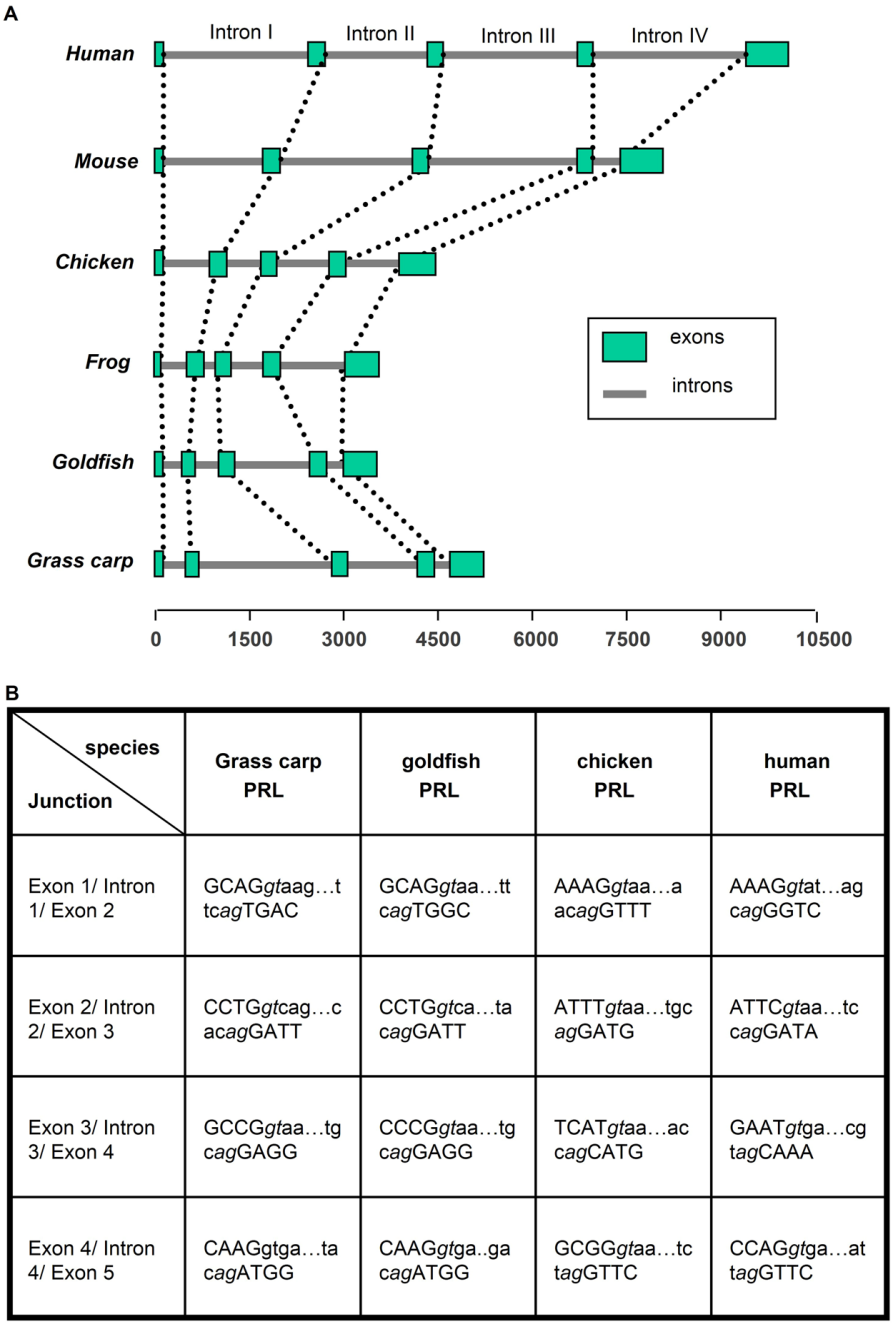
Genomic organization of PRL gene. (**A**) Comparison of the structures of vertebrate PRL genes. The grass carp PRL gene was aligned with that human, chicken, frog (xenopus) and goldfish homologous with respect to the ATG translation start codon at the 3′-end of exon 1. Boxes and lines represent exons and introns, respectively. The scale bar at the bottom represents the distance in kb with respect to the translation start codon ATG. (**B**) DNA sequences at the intron/exon junctions in different PRL genes. Exon sequences are shown in capital letters and the intron sequences are shown in lower case. The consensus intron splice donor and acceptor sequences are shown in italics.

Based on the genomic organization of PRL gene reported in mammals, chicken and frog, primers flanking the intron/exon junctions were designed according to the known sequences of grass carp PRL cDNA (gene bank No: EU074210). After that, intron trapping was performed using genomic DNA as the template to pull out the DNA sequence for intron I, II, III, and IV, and the associated coding exons (Supplementary Fig. 1). Using primers located in the 5′ UTR of grass carp PRL gene, a 1156 bp 5′ promoter of PRL gene was also isolated by genomic walking techniques (Supplementary Fig. 2). The PRL gene obtained (excluding 5′ promoter) is about 5 kb in size with 5 exons and 4 introns. The size of exon I, II, III, IV and V is 84 bp, 122 bp, 108 bp, 183 bp and 671 bp in size, respectively. Their intervening introns, namely intron I to IV, are 204 bp, 2370 bp, 1130 bp, and 153 bp, respectively. When compared with that of other vertebrates, including mammals, birds and amphibian, the genomic organization of PRL gene in grass carp is well conserved (Fig. 1A). Similar to other vertebrates, the intron I of grass carp PRL gene interrupts the coding sequence immediately after the ATG translation start codon and the C-terminal amino acid codons together with the entire 3′-UTR are encoded in the last exon. Apparently, the sizes of intron I, II, and III in mammalian PRL gene tend to be notably larger than that of the corresponding regions in non-mammalian species. The size of intron II of grass carp PRL is even bigger (2370 bp). Furthermore, sequence analysis of the mRNA splicing sites located in the “exon/intron” junctions of PRL genes from various classes of vertebrates reveals that they are all compile with the ‘GT/AG” rule as described previously^22^ (Fig. 1B), suggesting that the mechanisms for removing spliceosomal introns are well conserved in PRL genes. In the 5′ promoter obtained by genome walking, homology site search using TESS program (http://www.cbil.upenn.edu/cgi-bin/tess/tess) has revealed the presence of two TATA boxes located at position −20 and −261 upstream of the transcription initiation site. Multiple cis-acting elements including the binding sites for Pit1, Sp1, AP-1, NF-kappaB, GATA1, El2, HNF-3B, TBP, Oct1, and C/EBPalpha can also be identified in the various locations spreading along the entire length of the grass carp PRL promoter (Supplementary Fig. 2).

### Determination of the Transcriptional Start Site

**Figure 2:**
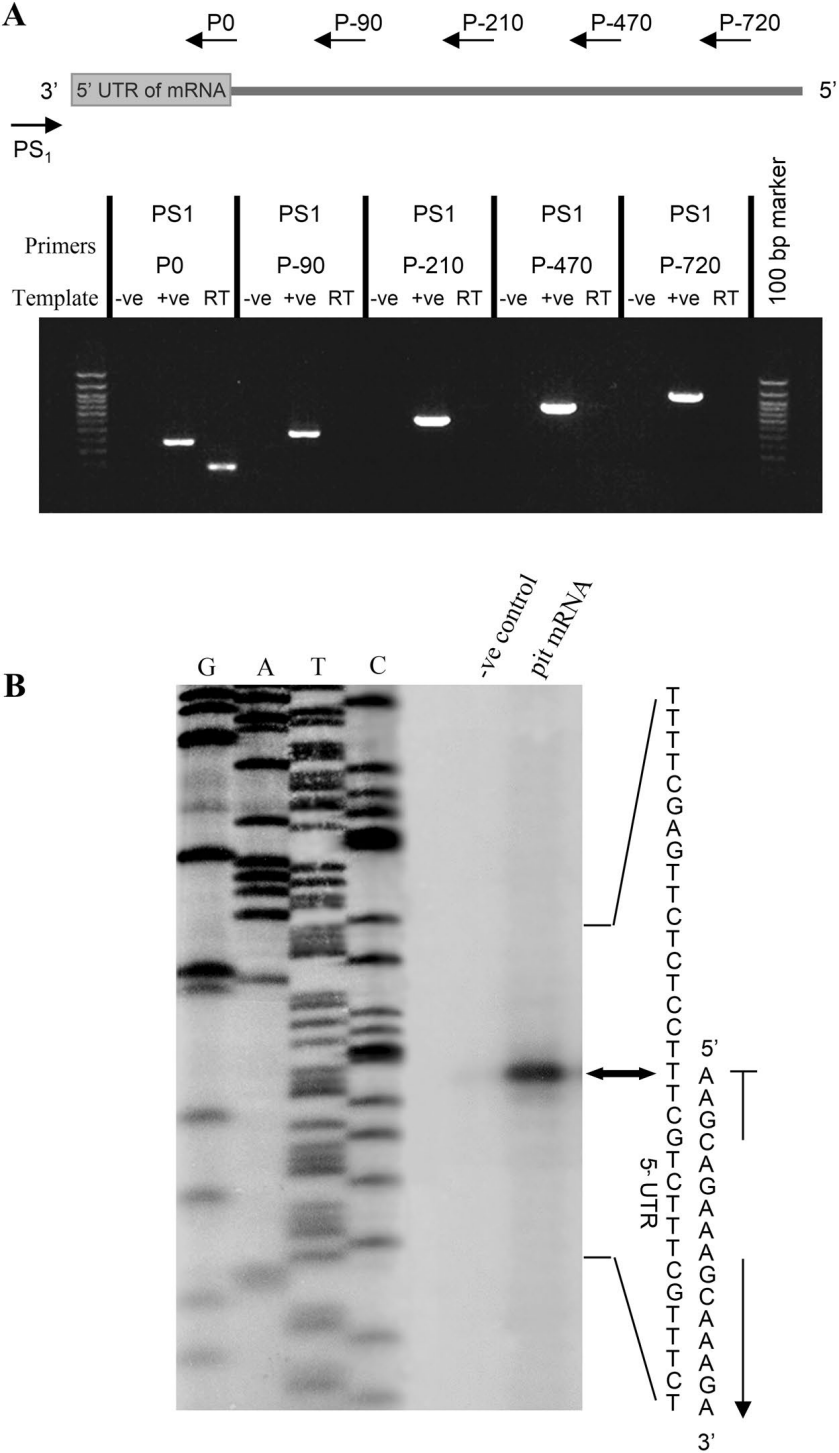
Mapping of the transcription start site (TSS) of PRL gene in grass carp. (**A**) Primer scanning to determine the region containing TSS. PCR amplification was performed to scan the possible region using downstream primer PS1 as the anchor primer combined with series upstream primers, respectively. In the PCR reactions, reverse transcribed cDNAs from carp pituitary total RNA were used as the template and parallel PCR with genomic DNA was used as the positive control. PCR with DDW as a template was used as the negative control (−ve). (**B**) Primer extension to identify the position of transcription start site. Total RNA (20 μg, lane 3) were prepared from steady-state incubated grass carp pituitary cells and hybridized with the [γ-32P] – labeled extension primer PE1. DNA sequencing ladder with PRL promoter (left lanes C, T, A, and G) was also performed to determine the position of reverse transcribed cDNAs. The primer extension samples together with sequencing ladders were size-fractionated with in an 8% polyacrylamide gel to identify the TSS.

To delineate the promoter region of grass carp PRL gene, primer scanning followed by primer extension was performed to define the location of the transcription initiation site of the newly cloned PRL promoter. In this study, primer scanning was first used to map the coarse location of the transcription initiation site while primer extension was later conducted to delineate the precise position of the junction between the 5′ promoter and 5′ UTR (as “+1” of grass carp PRL gene). For primer scanning, the primer PS1 located in the 3′ downstream region of 5′ UTR was used as the anchor primer for RT-PCR with the 5′ upstream primers P(−720), P(−470), P(−210), and P(−90) located at position −720, −470, −210, and −90 of the grass carp PRL promoter, respectively (Fig. 2A). As a control for the quality of the pituitary RT samples prepared, RT-PCR with the anchor primer PS1 and the 5′ upstream primer P0 located in the distal end of 5′UTR was also conducted. In this case, a 130 bp PCR product was consistently observed for RT-PCR with PS1 and P0, confirming that the quality of RT sample was appropriate for primer scanning. In this experiment, however, no PCR products could be detected in the RT-PCR using upstream primers located in position −720 to −90, suggesting that the transcription initiation site should be located within the sequence between position −90 and the 5′ end of the primer P0. The absence of PCR products in RT-PCR with other upstream 5′ primers could not be due to limitations in PCR reaction as the positive control with genomic DNA consistently produced PCR products of the expected sizes. The PCR product using genomic DNA as the template was 334 bp larger than that based on RT sample due to the inclusion of intron 1 in the sequence. Based on the information obtained, a new primer PE1 located in the distal end of 5′UTR was designed and end-labeled with ^32^P for primer extension using RNA samples prepared from the carp pituitary (Fig. 2B). With reference to the sequencing ladders, a single primer extension signal with the size of 83 bp was detected by autoradiography whereas no signal was produced in the “−ve” control without RNA loading. By comparing the sequencing ladders using grass carp PRL as a template, the transcription initiation site (defined as “+1”) was mapped to the “A” residue 53 bp upstream of the translation initiation site of the grass carp PRL cDNA. This location is also identified to the putative transcription initiation site predicted by the MacVector computer program (version 10.0) based on sequence analysis and alignment with PRL genes reported in other species.

### Deletion, mutagenesis and truncation analysis of PRL promoter activity in αT3-1 cells

**Figure 3:**
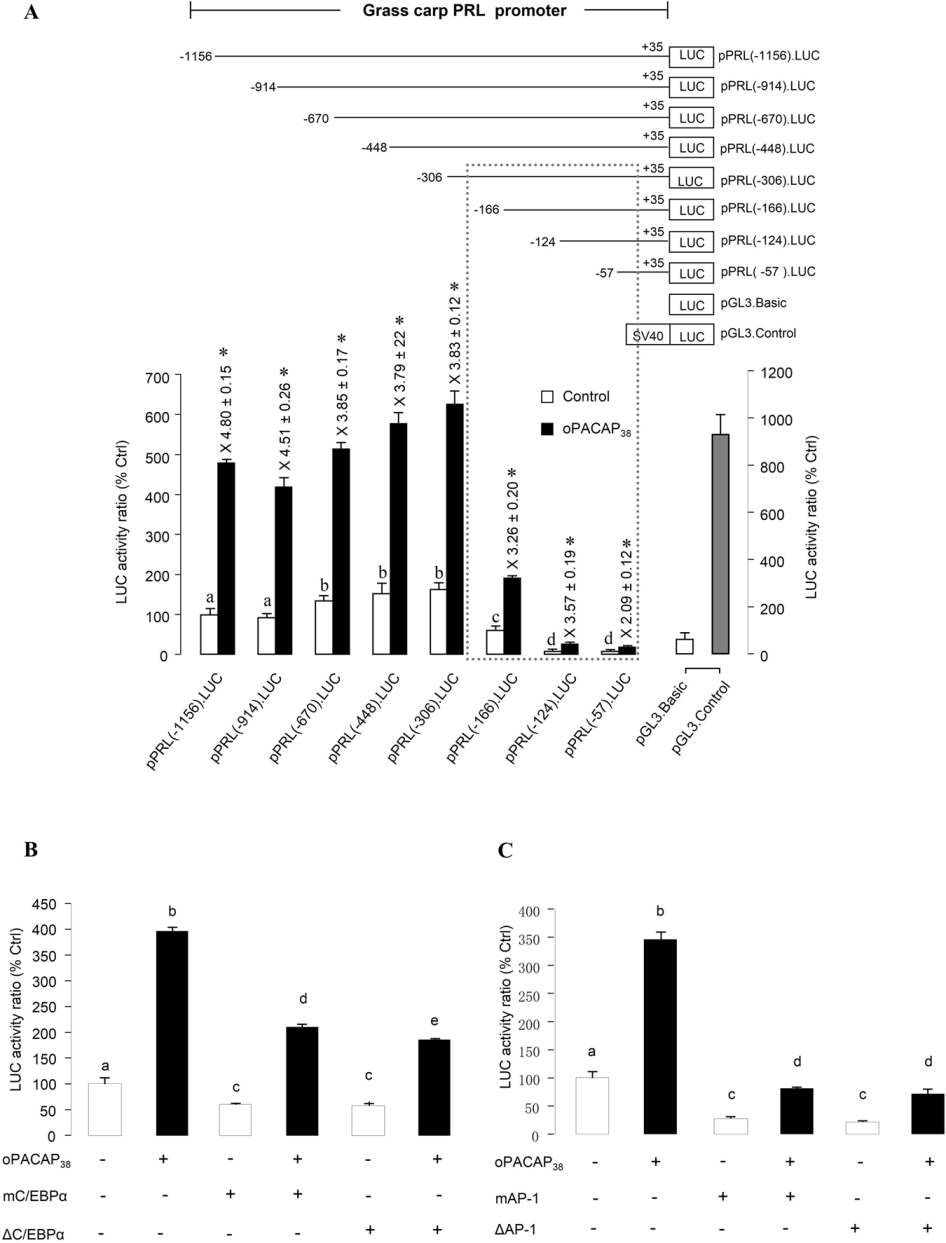
Deletion, mutagenesis and truncation analysis of PRL promoter activity in αT3-1 cells. (**A**) Deletion analysis of the oPACAP_38_-induced PRL promoter activity. The upper panel is the schematic diagram for pPRL.Luc deletion constructs with decreasing lengths of PRL promoter from position −1156 bp to −57 bp. The lower panel depicts PACAP effect on the luciferase activity of PRL promoter with serial deletions in the 5′ end. (**B**) Mutagenesis and truncation analysis on the role of C/EBPalpha (located at −226 to −217 of pPRL(−306)) in oPACAP_38_-induced PRL promoter activity. (**C**) Mutagenesis and truncation analysis on the role of AP1 (located at −136 to −130 of pPRL(−166))in oPACAP_38_-induced PRL promoter activity. (Mutation construct for C/EBPα: mC/EBPα; mutation construct for mAP-1: mAP-1; truncation construct for C/EBPα: ΔC/EBPα; truncation construct for AP-1: ΔAP-1) After transfection with the deletion/mutation/truncation constructs of PRL promoter, αT3-1 cells were challenged with PACAP (10 nM) for 24 hrs. Parallel transfections with the promoterless pGL3.Basic and pGL3.Control carrying a pSV40 promoter were also conducted as the negative and positive control, respectively. Data of relative firefly luciferase expression (mean ± SEM) (n = 4) are presented with percentage of control by conversing the ratio of firefly and renilla luciferase. For deletion analysis, the significant increase in luciferase activity expression with respect to the corresponding control is denoted by an asterisk (P < 0.05, Student’s t Test). Significant difference p < 0.05 (ANOVA followed by Fisher’s LSD Test) between the basal level of each deletion construct is denoted by different letters. For the mutagenesis and truncation analysis, different letters denote a significant difference at p < 0.05 (ANOVA followed by Fisher’s LSD Test).

To test the functionality of the grass carp PRL promoter, a series of deletion constructs were prepared by subcloning decreasing lengths of 5′ promoter sequence of PRL gene into the luciferase-expressing reporter pGL3.Basic. These 5′ deletion constructs were transiently transfected into the rat pituitary cell line αT3-1 and the stimulatory effect on luciferase activity expression was monitored after 24-hr treatment with ovine PACAP (100 nM). In this study, the upstream promoter of the carp PRL gene, including pPRL(−1156), pPRL(−914), pPRL(−670), pPRL(−448) and pPRL(−306), acted as strong promoters in αT3-1 cells. In the absence of drug treatment, basal luciferase activity was elevated by removing the promoter sequence from −914 to −670, suggesting that inhibiting element(s) may be present within this region. The promoter activity was significantly decreased by further deletion, either between pPRL(−306) and pPRL(−166) and or between pPRL(−166) and pPRL(−124), but not shown in the region between pPRL(−124) and pPRL(−57) (Fig. 3A). These results suggest that the proximal promoter region of grass carp PRL gene downstream of position −306 may contain the major cis-acting elements responsible for PACAP stimulation of PRL expression. Subsequently, mutagenesis and truncation analysis were performed to investigate the role of transcription sites in PRL promoter activity. The results showed that not only the promoter activity stimulated by PACAP but also the basal activity was significantly suppressed by the mutagenesis and truncation of the C/EBPalpha transcription site from −226 to −217 (Fig. 3B) and the AP-1 transcription site from −136 to −130 (Fig. 3C). These results coincided well with the results in the deletion analyses, in which both the basal and stimulated (by PACAP) activities were suppressed in the construct (−166; no C/EBPalpha) and further in the construct (−124; no AP1).

### PACAP-stimulated Prolactin Promoter Activity via PAC1 receptor in αT3-1 Cells

**Figure 4:**
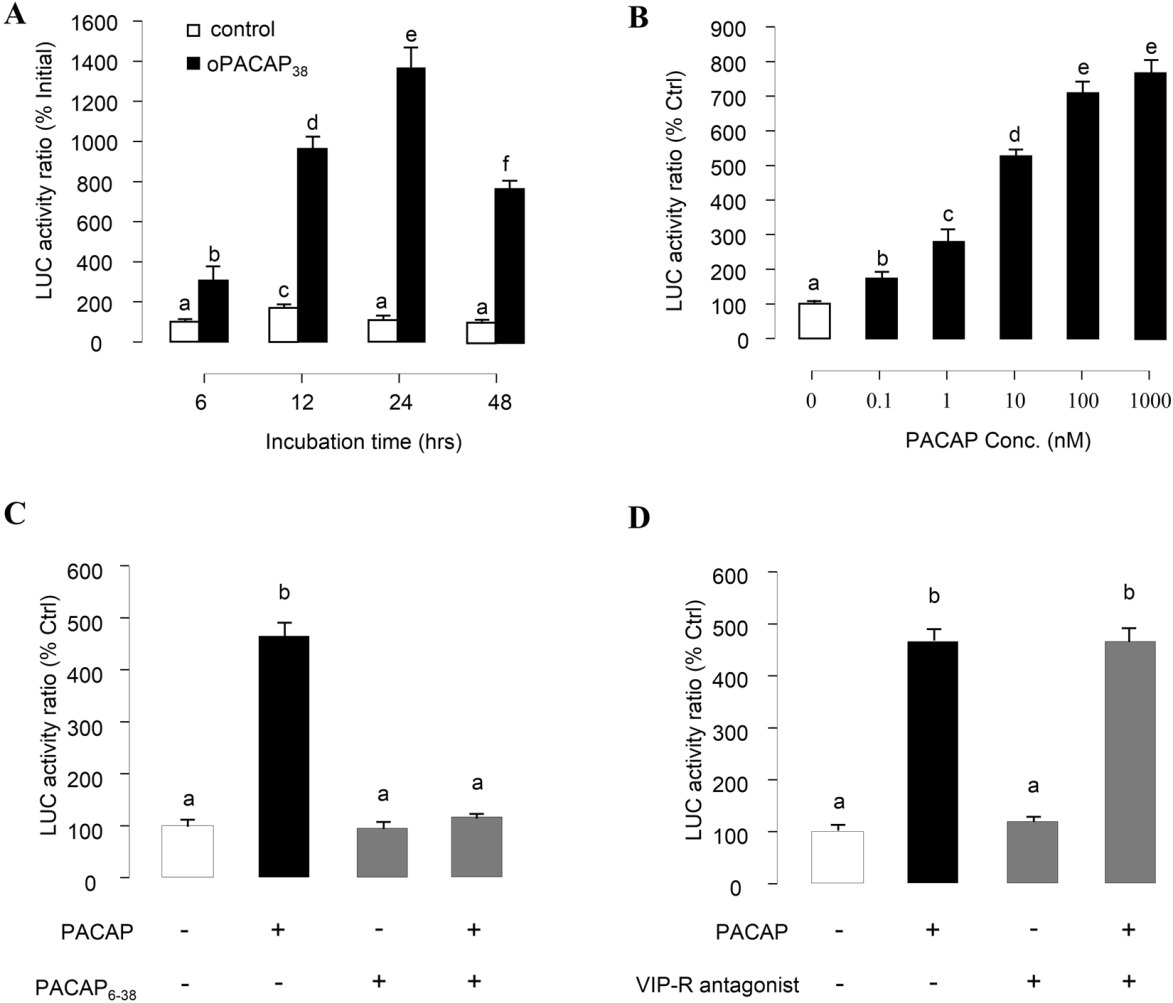
Receptor specificity of PACAP-induced PRL promoter activity in αT3-1 Cells. αT3-1 cells were transiently transfected with pPRL(−1156).LUC for 6 h by using lipofectamine. The cells were then cultured for 18 hr recovery before drug treatment. (**A**) Time course analysis on the effect of oPACAP_38_ (6–48 hrs) on grass carp PRL promoter activity in αT3-1 cells. (**B**) αT3-1 cells over-expressed pPRL(−1156).LUC were treated for 24 hrs with increasing doses of oPACAP_38_. Effects of PACAP and VIP antagonists on PACAP-induced PRL mRNA expression were also investigated. In these experiments, αT3-1 cells over-expressed pPRL(−1156).LUC were challenged with oPACAP_38_ (10 nM, 24 hr) in the presence or absence of (**C**) the PACAP antagonist PACAP6-38 (10 nM) or (**D**) VIP antagonist (4-Cl-D-Phe6, Leu17)VIP (“VIP-R antagonist”, 100 nM). After drug treatment, cell lysate was prepared for dual-luciferase measurement. Data presented were expressed as percentage of control by conversing the ratio of firefly and renilla luciferase in the same sample. Data presented are expressed as mean ± SEM (n = 4) and different letters denote a significant difference at p < 0.05 (ANOVA followed by Fisher’s LSD Test).

Since pPRL(−1156).Luc consistently produced high levels of both basal and PACAP-induced luciferase activity, the construct was routinely used in our transfection studies for PACAP-stimulated PRL promoter activity. In this case, PRL luciferase activity expressed in αT3-1 cells could be evaluated in a time-dependent manner by ovine PACAP_38_ (100 nM) with the peak response occurred at 24 hr after the initiation of drug treatment (Fig. 4A). By fixing the duration of drug treatment at 24 hr, increasing concentrations of PACAP (0.1–1000 nM) was found to up-regulate luciferase activity expression in a dose-dependent manner with the maximal response observed at 1000 nM and EC_50_ value at 4.5 nM (Fig. 4B). Consistently, the stimulatory effect on PRL promoter activity was also observed in αT3-1 cells incubated with increasing doses of grass carp PACAP_38_ (Supplementary Fig. 3). To further confirm the involvement of PAC1 receptor in PACAP-induced PRL promoter activity, αT3-1 cells were treated with oPACAP_38_ (10 nM) with/without simultaneous treatment with the PACAP antagonist PACAP_6–38_ (100 nM, Fig. 4C) or VIP antagonist (4-Cl-d-Phe6, Leu17) VIP (100 nM, Fig. 4D). In the result, oPACAP_38_-induced PRL promoter acvtivity was blocked by the co-treatment with the PACAP antagonist PACAP_6–38_, but not the VIP antagonist (4-Cl-d-Phe6, Leu17)VIP.

### cAMP/PKA Pathway in PACAP Induction of PRL Promoter Activity

**Figure 5:**
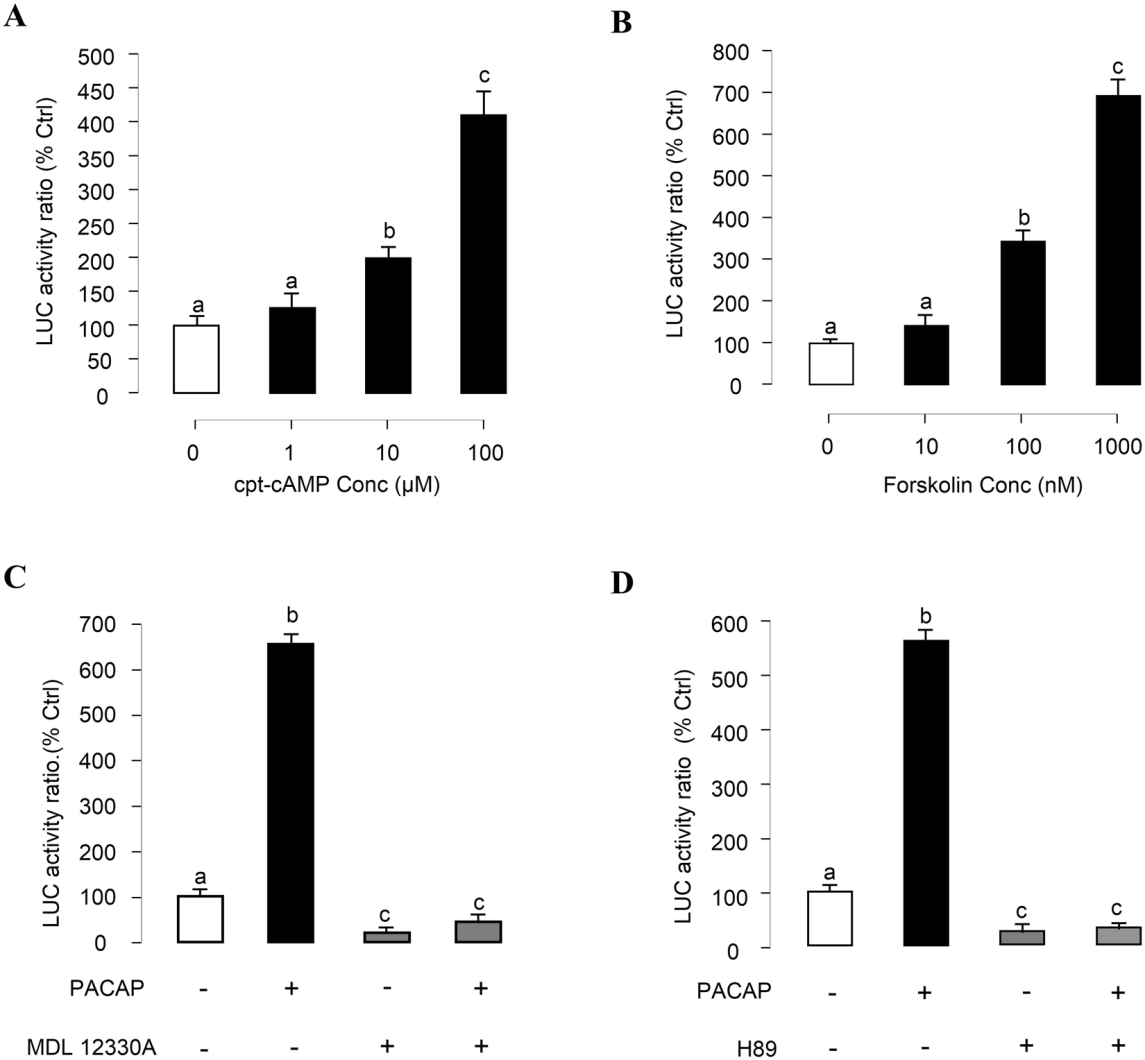
Functional role of cAMP/PKA pathway in PACAP stimulation of PRL promoter activity in αT3-1 Cells. αT3-1 cells were transiently transfected with pPRL(−1156).LUC for 6 h by using lipofectamine. After 18 h recovery, the cells were incubated with respective drugs. (**A**) αT3-1 cells over-expressed pPRL(−1156).LUC were treated for 24 hrs with increasing doses of cpt-cAMP (1–100 μM). (**B**) αT3-1 cells over-expressed pPRL(−1156).LUC were treated for 24 hrs with increasing doses of Forskolin (10–1000 nM). Effects of cAMP/PKA inhibitors on PACAP-induced PRL mRNA expression were then investigated. αT3-1 cells over-expressed pPRL(−1156).LUC were challenged with oPACAP_38_ (10 nM, 24 hr) in the presence or absence of (**C**) AC inhibitor MDL12330A (10 μM) or (**D**) PKA blocker H89 (10 μM). After drug treatment, cell lysate was prepared for dual-luciferase measurement. Data presented were expressed as percentage of control by conversing the ratio of firefly and renilla luciferase in the same sample. Data presented are expressed as mean ± SEM (n = 4) and different letters denote a significant difference at p < 0.05 (ANOVA followed by Fisher’s LSD Test).

Given that cAMP production in αT3-1 cells can be induced by PACAP via activation of PAC1 receptors^23^ and our recent studies in grass carp pituitary cells have shown that PACAP can trigger PRL mRNA expression via activation of pituitary PAC1 receptors^21^, the involvement of the cAMP-dependent cascades in PACAP induced PRL promoter activity was investigated. As a first step, αT3-1 cells were treated for 24 hrs with increasing levels of the membrane permeable cAMP analog cpt-cAMP (1–100 μM) and adenylate cyclase (AC) activator Forskolin (10–1000 nM). In these experiments, luciferase activity expression in αT3-1 cells was dose-dependently increased by cpt-cAMP (Fig. 5A) and Forskolin (Fig. 5B), respectively. In parallel studies, luciferase activity expression was consistently induced by 24-hr treatment with ovine PACAP_38_ (10 nM) and this stimulatory effect could be totally abolished by simultaneous treatment with the AC inhibitor MDL12330A (10 μM, Fig. 5C) or the PKA inhibitor H89 (10 μM, Fig. 5D). In the same experiments, basal levels of luciferase activity detected in αT3-1 cells were also markedly suppressed by the pharmacological inhibitors for AC and PKA, respectively.

### Ca^2+^-dependent Cascades in PACAP-induced PRL Promoter Activity

**Figure 6:**
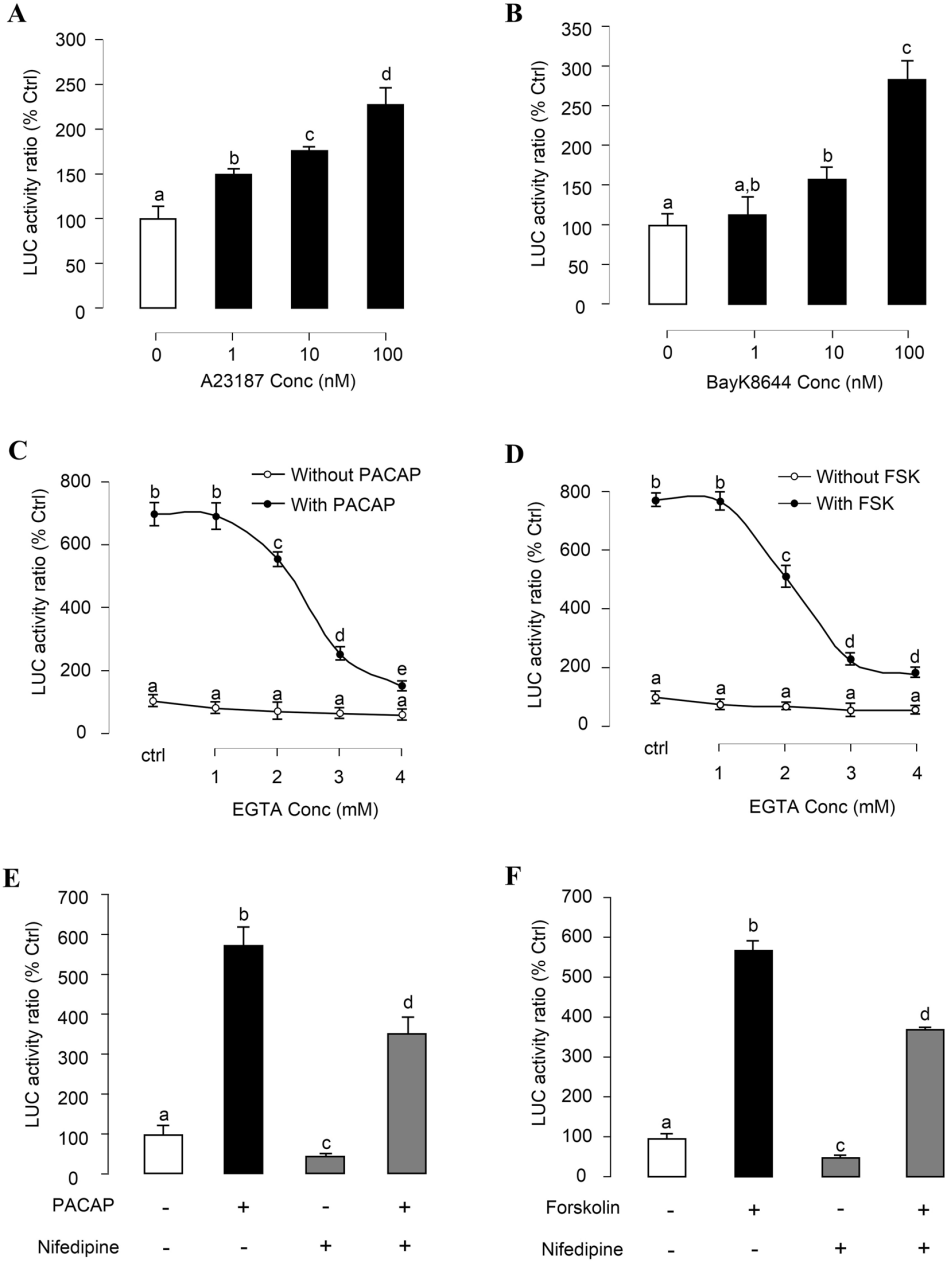
Ca^2+^-dependent of PACAP- and Forskolin-induced PRL promoter activity in αT3-1 Cells. αT3-1 cells were transiently transfected with pPRL(−1156).LUC for 6 h by using lipofectamine. After 18 h recovery, the cells were incubated with respective drugs. The cells were treated for 24 hr with increasing doses of (**A**) Ca^2+^ ionophore A23187 or (**B**) L-type VSCC activator Bay K8644. Inhibiting extracellular Ca^2+^ entry on PRL mRNA expression in carp pituitary cells was examined. In this study, αT3-1 cells over-expressed pPRL(−1156).LUC were treated with (**C**) oPACAP_38_ (10 nM, 24 hrs) or (**D**) Forskolin (100 nM, 24 hrs) in the Ca^2+^-free medium (with different doses of EGTA). Further, αT3-1 cells over-expressed pPRL(−1156).LUC were treated with (**E**) oPACAP_38_ (10 nM, 24 hrs) or (**F**) Forskolin (100 nM, 24 hrs) in the presence or absence of L-type VSCC blocker nifedipine (10 μM). After drug treatment, cell lysate was prepared for dual-luciferase measurement. Data presented were expressed as percentage of control by conversing the ratio of firefly and renilla luciferase in the same sample. Data presented are expressed as mean ± SEM (n = 4) and different letters denote a significant difference at p < 0.05 (ANOVA followed by Fisher’s LSD Test).

**Figure 7:**
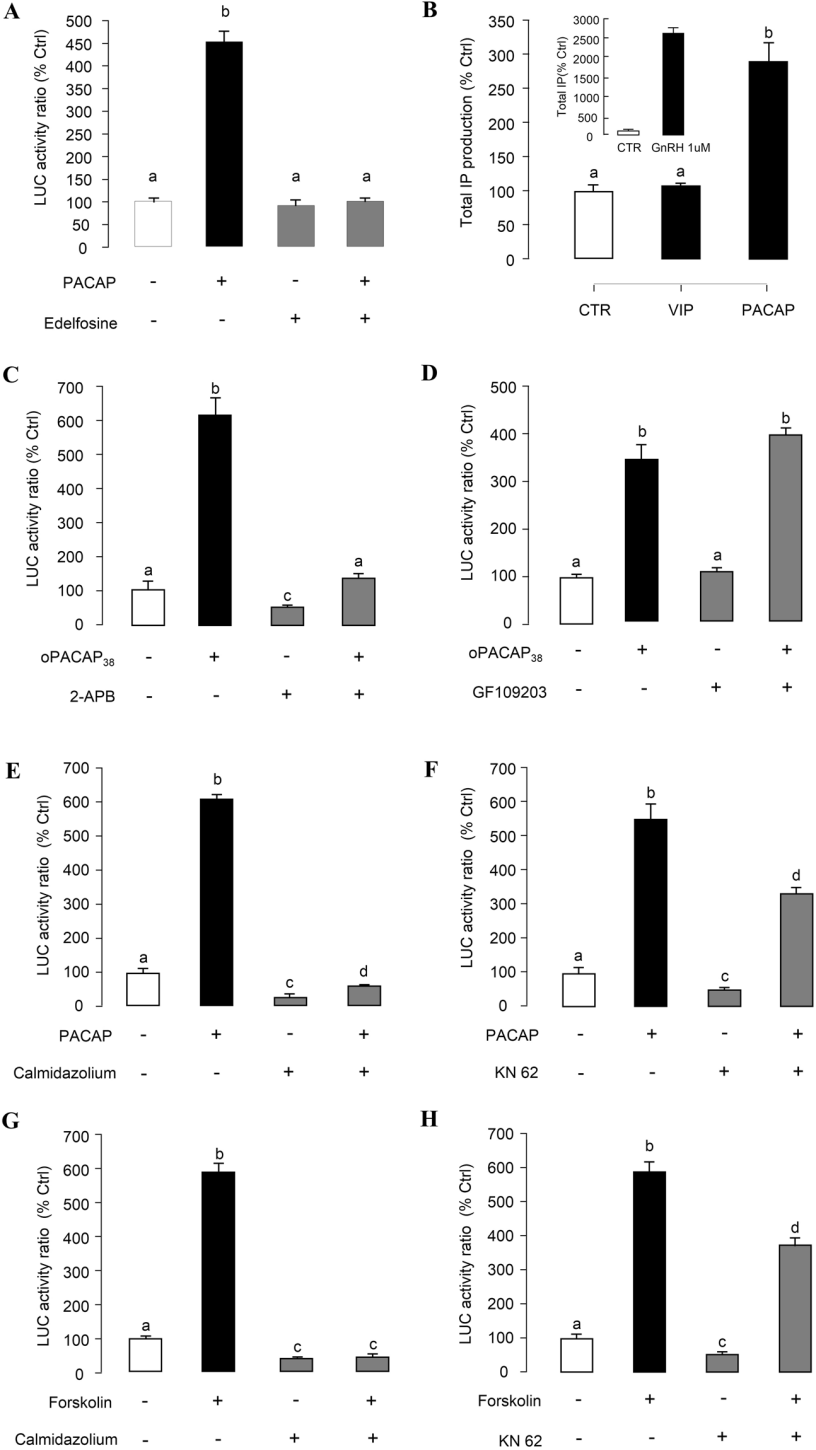
Functional role of the PLC/IP3/PKC and CaM/CaMK-II cascades in PACAP- and Forskolin-induced PRL promoter activity. αT3-1 cells were transiently transfected with pPRL(−1156).LUC for 6 h by using lipofectamine. After 18 h recovery, the cells were incubated with respective drugs. (**A**) αT3-1 cells over-expressed pPRL(−1156).LUC were treated with oPACAP_38_ (10 nM, 24 hrs) in the presence or absence of PLC inhibitor Edelfosine (20 μM). (**B**) Transfected αT3-1 cells were labeled with 2 μCi/well of myo-[3 H] inositol (DuPont/NEN) in myo-inositol free DMEM medium containing 10% fetal bovine serum and then treated with oPACAP_38_, VIP, and GnRH for 45 mins. The total IP production were analyzed by detecting the radio-labelled inositol incorporation. Then, αT3-1 cells over-expressed pPRL(−1156).LUC were treated with oPACAP_38_ (10 nM, 24 hrs) in the presence or absence of (**C**) IP3 receptor inhibitor 2-APB (100 μM), (**D**) PKC inhibitor GF109203 (1 μM), (**E**) CaM antagonist calmidazolium (1 μM) and (**F**) CaM KinaseII inhibitor KN62 (5 μM). In parallel, the transfected cells were challenged with Forskolin (100 nM, 24 hrs) in the presence or absence of CaM antagonist calmidazolium (**G**, 1 μM) and CaM KinaseII inhibitor KN62 (**H**, 5 μM). After drug treatment, cell lysate was prepared for dual-luciferase measurement. Data presented were expressed as percentage of control by conversing the ratio of firefly and renilla luciferase in the same sample. Data presented are expressed as mean ± SEM (n = 4) and different letters denote a significant difference at p < 0.05 (ANOVA followed by Fisher’s LSD Test).

To test the role of Ca^2+^-dependent pathway in PACAP induction of PRL promoter activity, [Ca^2+^]e influx was induced in αT3-1 cells by 24-hr treatment with increasing doses of the Ca^2+^ ionophore A23187 (1–100 nM, Fig. 6A) and votage-sensitive Ca^2+^ channel (VSCC) activator Bay K8644 (1–100 nM, Fig. 6B). These drug treatments were both effective in stimulating luciferase activity expression in αT3-1 cells. To clarify the functional link between cAMP- and Ca^2+^-dependent pathway, the stimulatory effects of PACAP and Forskolin on luciferase activity were tested with gradual removal of [Ca^2+^]e using increasing doses of the Ca^2+^ chelator EGTA (1–4 mM). In this case, PACAP (Fig. 6C) and Forskolin (Fig. 6D) inductions of luciferase activity expression could be suppressed dose-dependently by EGTA treatment. In parallel experiments, blocking [Ca^2+^]e entry via VSCC using the VSCC inhibitor nifedipine (10 μM) was also effective in attenuating PACAP- and Forskolin-induced luciferase activity expression in αT3-1 cells (Fig. 6E,F). In our recent studies in grass carp pituitary cells, IP3-sensitive Ca^2+^ stores also play a role in PACAP-induced PRL mRNA expression^21^. Here similar results were shown in Fig. 7B, PACAP but not VIP treatment could enhance total IP level in αT3-1 cells. GnRH (1 μM) treatment, which was used as a “+ve” control in this experiment, also significantly increased the total IP level in αT3-1 cells (Fig. 7B, inset). Moreover, the inhibitors for PLC (Edelfosine, 20 μM, Fig. 7A; U73122, 10 μM, Supplementary Fig. 4A), and IP3 receptor (2-APB, 100 μM, Fig. 7C) effectively abolished the stimulatory effect of PACAP on the luciferase activity. Although PLC is well-documented to trigger PKC activation together with IP3 production, co-treatment with the PKC inhibitor GF109203 (1 μM) was not effective in altering PACAP induction of luciferase activity expressed in αT3-1 cells (Fig. 7D). To elucidate the downstream signaling of the Ca^2+^-dependent cascades, the stimulatory effect of PACAP on PRL promoter activity was tested with CaM antagonism by calmidazolium (1 μM, Fig. 7E), W7 (30 μM, Supplementary Fig. 4B), W13 (100 μM, Supplementary Fig. 4C) and CaMK-II blockage by KN62 (5 μM, Fig. 7F). In this case, basal luciferase activity expression and the stimulatory action of PACAP were both suppressed/totally inhibited by the drug treatment. Similar results were also obtained by substituting Forskolin (100 nM) for PACAP (10 nM) as the stimulant for luciferase activity expression in αT3-1 cells (Fig. 7G,H).

### MAPK and PI3K Cascades in PACAP-induced PRL Promoter Activity

**Figure 8:**
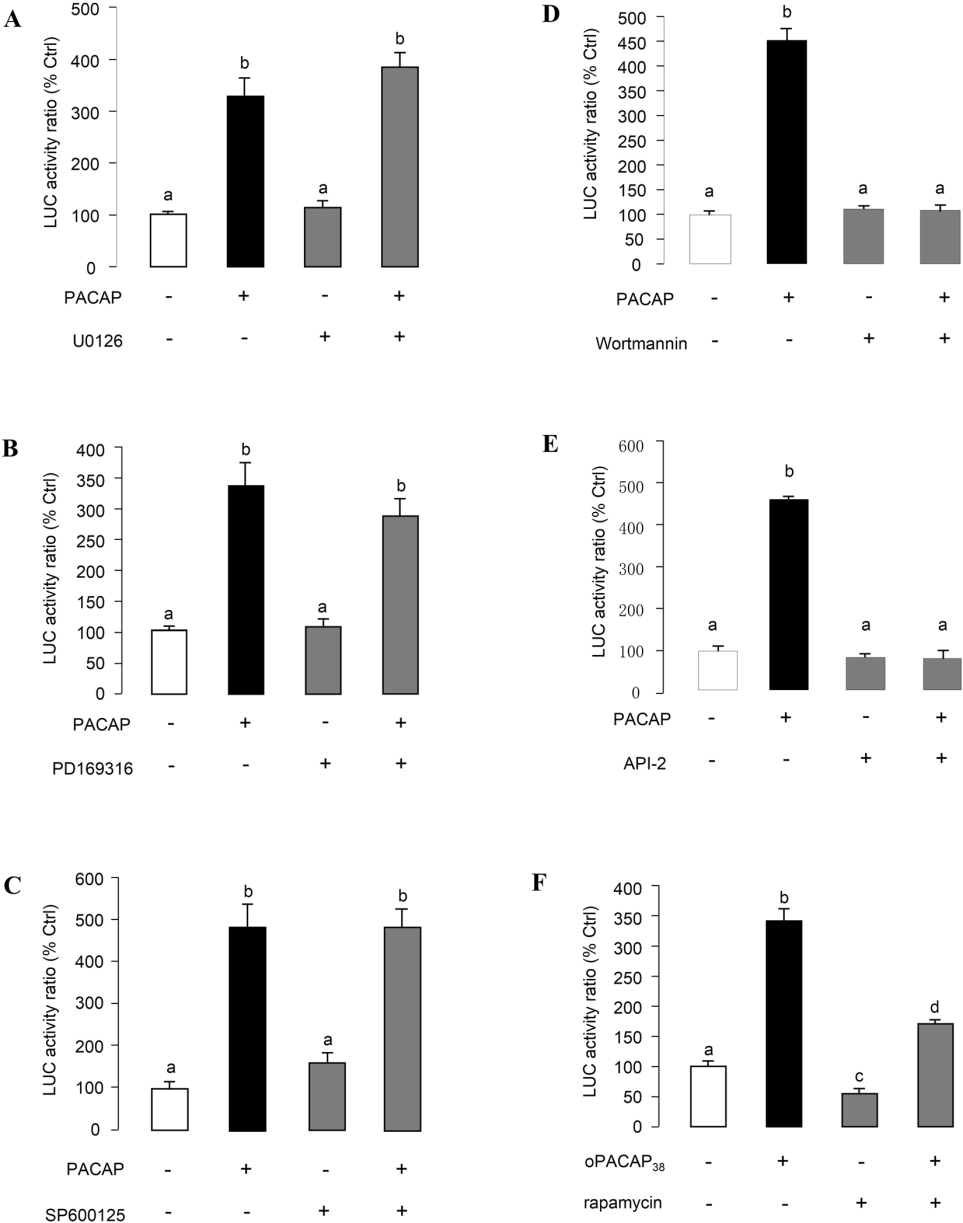
Functional role of MAPK cascades and PI3K/Akt/P70S6K pathway in PACAP-induced PRL promoter activity. αT3-1 cells were transiently transfected with pPRL(−1156).LUC for 6 h by using lipofectamine. After 18 h recovery, the cells were incubated with respective drugs. In this study, αT3-1 cells over-expressed pPRL(−1156).LUC were treated with oPACAP_38_ (10 nM, 24 hrs) in the presence or absence of (**A**) ERK1/2 inhibitor U0126 (10 μM), (**B**) p38MAPK inhibitor PD169316 (10 μM), (**C**) JNK inhibitor SP600125 (10 μM), (**D**) PI3K inhibitor Wortmannin (10 nM), (**E**) P_70_^S6K^ inhibitor rapamycin (20 nM) and (**F**) Akt inhibitor API-2 (10 uM). After drug treatment, cell lysate was prepared for dual-luciferase measurement. Data presented were expressed as percentage of control by conversing the ratio of firefly and renilla luciferase in the same sample. Data presented are expressed as mean ± SEM (n = 4) and different letters denote a significant difference at p < 0.05 (ANOVA followed by Fisher’s LSD Test).

Given that PACAP has been reported to activate MAPK (e.g., in human SH-SY5Y neuroblastoma cells)^24^, and PI3K-dependent cascades (e.g., in human neutrophils)^25^, the functional role of MAPK and PI3K in PACAP-induced grass carp promoter activity were also examined. In αT3-1 cells, both basal and PACAP (10 nM)-induced luciferase activity expression was not affected by simultaneous treatment with the P_42/44_^MAPK^ (or Erk1/2) inhibitor U0126 (10 μM, Fig. 8A), P_38_^MAPK^ inhibitor PD169316 (10 μM, Fig. 8B), and JNK inhibitor SP600125 (10 μM, Fig. 8C). However, parallel treatment with the PI3K inhibitor LY294002 (10 μM, Supplementary Fig. 4D) and Wortmanin (10 nM, Fig. 8D) was effective in reducing basal as well as PACAP induction of luciferase activity. To further elucidate the downstream signaling events after PI3K activation, αT3-1 cells were treated with PACAP (10 nM) in the presence of the _70_^S6K^ inhibitor rapamycin (20 nM, Fig. 8E) or the Akt/PKB inhibitor API-2 (20 μM, Fig. 8F). In this case, PACAP-induced luciferase activity expression could be attenuated by rapamycin and API-2, suggesting both p70S6K and Akt are involved in the PACAP-induced PRL promoter activity.

### CREB in PACAP Induction of PRL Promoter Activity

**Figure 9:**
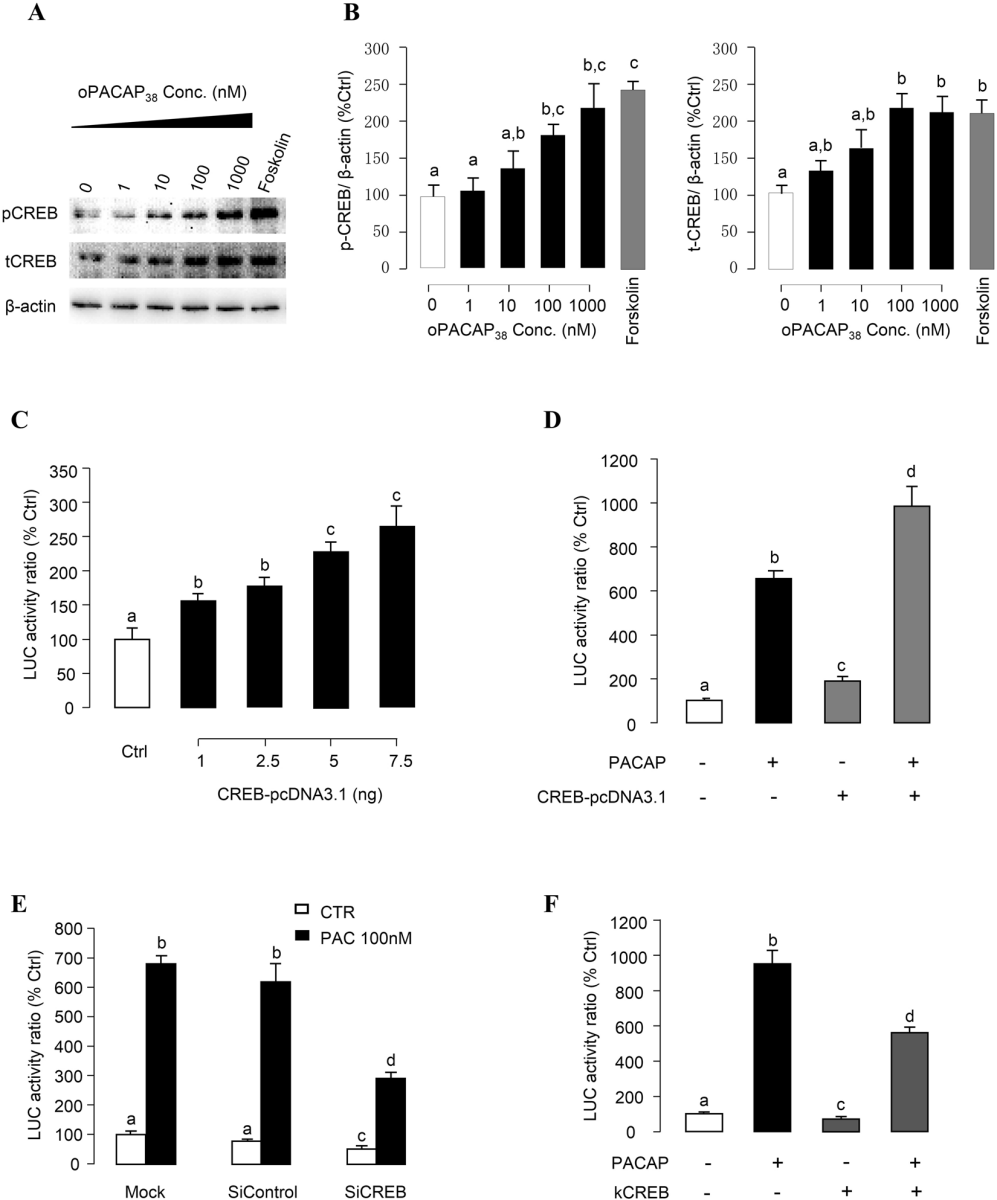
Functional role of CREB in PACAP-induced PRL promoter activity. (**A**) Effect of oPACAP_38_ on CREB phosphorylation and total production in grass carp pituitary cells. Pituitary cells were treated with oPACAP_38_ with the indicated doses or Forskolin (1 μM) for 24 hrs. After drug treatment, cell lysate was harvested for Western blot analysis on pCREB and tCREB. β-actin was blotted as internal control. (**B**) Statistical charts of the western blot results quantified by Image J software. (**C**) αT3-1 cells were co-transfected with pPRL(−1156).LUC and increasing doses of grass carp CREB expression vector CREB-pcDNA3.1 for 24 hrs. (**D**) αT3-1 cells transfected with pPRL(−1156).LUC were treated by oPACAP_38_ (10 nM, 24 hrs) in the presence or absence of CREB-pcDNA3.1 over-expression. (**E**) αT3-1 cells transfected with pPRL(−1156).LUC were treated by oPACAP_38_ (10 nM, 24 hrs) in the presence or absence of siRNA for CREB or siRNA control. (**F**) αT3-1 cells transfected with pPRL(−1156).LUC were treated by oPACAP_38_ (10 nM, 24 hrs) in the presence or absence of dominant negative CREB mutant kCREB overexpression. For transfection experiments, after drug treatment, cell lysate was prepared for dual-luciferase measurement. Data presented were expressed as percentage of control by conversing the ratio of firefly and renilla luciferase in the same sample. Data presented are expressed as mean ± SEM (n = 4) and different letters denote a significant difference at p < 0.05 (ANOVA followed by Fisher’s LSD Test).

It has been shown that CREB plays an important role for the genomic actions mediated through the cAMP/PKA or Ca^2+^/CaM-dependent pathways by binding to CRE sites in target gene promoters^26–28^. In grass carp pituitary cells, PACAP treatment significantly enhanced CREB phosphorylation and stimulated total CREB production (Fig. 9A,B), indicating that CREB may serve as the transcription factor mediating PACAP induction of grass carp PRL gene transcription. In αT3-1 cells, pPRL(−1156).Luc was cotransfected with increasing doses of the expression vector for grass carp CREB (1–7.5 ng), resulting in dose-dependent increase of luciferase activity expression after 24 hr incubation (Fig. 9C). Parallel studies with PACAP treatment also revealed that cotransfection with CREB expression vector (7.5 ng) could not only elevate basal but also enhance the stimulatory effect of PACAP in luciferase activity expressed in αT3-1 cells (Fig. 9D). In contrast, PACAP-induced luciferase activity expression was significantly suppressed by CREB siRNA (Fig. 9E). Parallel transfection with siRNA control with scramble sequence was not effective in this regard. Further, both basal and PACAP-stimulated luciferase activity expressed in αT3-1 cells could be reduced by over-expression of kCREB (Fig. 9F), a domain negative mutant of CREB which can block CREB binding to CRE sites in target gene promoter^29^.

## Discussion

The newly cloned PRL gene spans 5 kb in size and consists of 5 exons and 4 introns. All the introns identified have been confirmed to start with “GT” and terminated with “AG”, which is consistent with the ‘GT/AG” rule^22^. The exon/intron junctions of the grass carp PRL gene is highly comparable to that of PRL genes reported for different classes of vertebrates, suggesting that the process involved in the splicing of individual introns from the primary transcripts of PRL is highly conserved during the evolution of vertebrates. The enlargement of intron I and III in mammalian PRL gene indicates that additional nucleotide sequence might have been recruited in these regions prior to the evolution of mammals. The size of exon II increases from teleosts to mammals mainly due to the additional nucleotide sequence between the signal peptide and the first helical domain in mammalian PRL gene. It is also worth mentioning that the genomic organization of PRL genes examined is also identical to that reported for GH gene in fish (i.e., with 5 exons and 4 introns), consistent with the consensus that PRL and GH are both evolved from a common ancestral gene^1^.

The transcription start site of grass carp PRL is located at 53 bp upstream of the transcription start site, highly comparable to the result of recent study in goldfish PRL gene^6^. Apparently, the PRL genes of the *cyprinus* family, including both grass carp and goldfish, tend to have a very short 5′ UTR region. Our results, however, are at variance with that reported for human PRL gene, in which two separate transcription start sites have been identified^30^, with one for pituitary expression located in the proximal promoter region while the other located in the distal end of the upstream region about 5 kb away from the pituitary start site and is responsible for placental expression of PRL gene. In the proximal promoter of grass carp PRL gene, a typical TATA box with the sequence “TATAAT” was found 20 bp upstream of the transcription start site. TATA box can also be identified in the promoter region of the goldfish PRL promoter^6^. Similar findings have been described in the PRL gene promoter in common carp^31^, salmon^32^ and sea bream^33^, etc. Apparently, the presence of TATA box in the proximal region of PRL gene is a common feature for fish models.

Recently, we found that PACAP can stimulate PRL secretion, protein synthesis and gene expression in grass carp pituitary cells via activation of PAC1 receptor^21^. The parallel increases in PRL protein production and PRL mRNA expression observed in our study raise the possibility that PACAP may stimulate PRL synthesis via up-regulation of PRL gene transcription. This hypothesis has been confirmed by the present study using αT3-1 cells as the host cells to examine grass carp PRL promoter activity. In our studies with αT3-1 cells, grass carp PRL promoter activity could be induced by PACAP treatment in a time- and dose-dependent manner and the PACAP responsive sequence was mapped to proximal promoter region downstream of position −306 of the grass carp PRL promoter. Within this region, consensus sequence for C/EBP alpha and AP-1 binding sites can be identified. C/EBP proteins possess domains that contain cAMP-inducible activities that are independent of direct phosphorylation by PKA^34,35^. In mammalian melanocytes, AP-1 is known to be induced by cAMP and leads to an increased tyrosinase gene expression^36^. The mechanisms of C/EBP alpha and AP-1 binding sites working on grass carp PRL promoter regulation and stimulatory effects induced by PACAP remain to be further clarified.

In primary pituitary cells, PRL production induced by PACAP via functional coupling of cAMP/PKA−, Ca^2+^/CaM−, and MAPK-dependent cascades^21^. At the transcription level, cAMP/PKA and Ca^2+^/CaM/CaM-II pathways are consistently involved in the PACAP regulation on PRL promoter activity. In mammalian cell models, e.g., in human SH-SY5Y neuroblastoma cells, functional coupling of MAPK (e.g., Erk1/2 and P_38_^MAPK^) with cAMP-dependent mechanism has been reported^24^. In GH3 cells, PACAP triggered ERK activation to stimulate PRL synthesis^37^. However, in our studies with αT3-1 cells, the inhibitors for Erk1/2, and P_38_^MAPK^ were not effective in altering both basal and PACAP-induced PRL promoter activity. It is intriguing that JNK signaling was not involved in the PACAP regulation on PRL transcription, distinct from the previous findings that PACAP stimulate PRL mRNA expression through JNK activation in grass carp pituitary cells^21^. Apparently, the MAPK cascades are not involved in PACAP-induced grass carp PRL gene transcription. This is similar with another finding that PACAP stimulates ERK activation in αT3–1 cells, which mainly contributed to the increased DNA synthesis and cell proliferation rather than transcriptional activation of the αGSU gene^38^. Moreover, PACAP stimulation of grass carp PRL gene transcription also involve the activation of PI3K/p70S6K pathway, which was not exhibited in PACAP-induced PRL mRNA expression in grass carp pituitary cells^21^. This inconsistency may be due to the different cell types used in the individual studies. Since PKA activation is known to induce p70S6K phosphorylation, e.g., in sertoli cells after FSH stimulation^39^, we do not exclude the possibility that functional coupling between cAMP/PKA and PI3K/p70S6K pathway may also occur in grass carp PRL gene expression.

CREB is a member of the leucine zipper transcription factors and well-known to be involved in the activation of cAMP-inducible genes^26^. In mammalian cell models, PACAP treatment has been reported to trigger CREB phosphorylation via cAMP/PKA^40^, cAMP/ERK^41^, and/or PLC/PKC^42^ pathways. Over-expression of a constitutively active mutant of CREB can activate rat PRL promoter activity via both CRE and non-CRE mediated mechanisms^43,44^. Expression of a dominant-negative form of CREB (MCREB) inhibited basal and Forskolin-induced PRL promoter activity and PRL mRNA expression in rat lactotrophs^45^. In this study, similar to the case of the rat PRL promoter, over-expression of grass carp CREB not only elevated basal but also enhanced PACAP-induced grass carp PRL promoter activity in αT3-1 cells. The stimulatory effect of PACAP on PRL promoter activation was significantly suppressed by CREB gene silencing using siRNA as well as by over-expression of a domain negative mutant of CREB, namely the kCREB. Moreover, in grass carp pituitary cells, PACAP can stimulate CREB phosphorylation and production. These results provide evidence that CREB activation is involved in PACAP induction of PRL gene transcription in grass carp. Since CREB is the downstream phosphorylation substrate for PKA, CaMK-II as well as other signaling kinases, it is conceivable that CREB may serve as the transcription factor mediating PACAP’s action on PRL gene via the cAMP, Ca^2+^ and PI3K signaling cascades^46^. Although CRE and/or CRE-like elements (CLE) have been identified in the 5′ promoter of PRL gene in mammals [e.g., rat]^47^ as well as in fish models [e.g., sea bream^33^ and common carp]^48^, consensus sequence for CRE could not be located in the grass carp PRL promoter by TESS site search. It raises the possibility that a non-conanical CRE(s) may be present in the grass carp PRL gene and mediates CREB transactivation of PRL promoter. In mammals, CREB can form heterodimers with transcription factors of the AP-1 family^49^. In PC12 cells, phosphorylated CREB was found to activate c-fos transcription^50^. Thus alternatively, the induction effect of CREB on PRL gene transcription may be mediated by other transcription factors.

**Figure 10:**
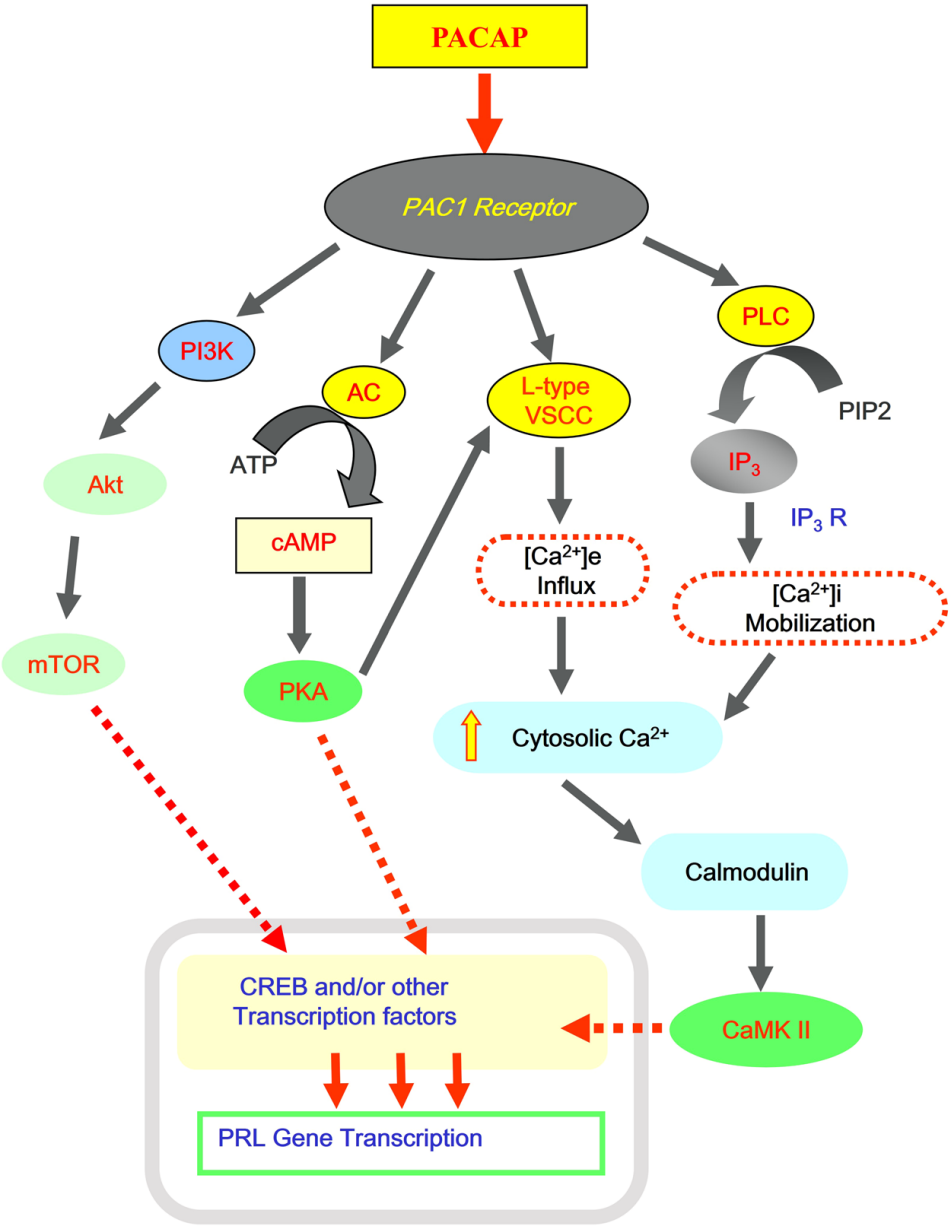
Summary of the signaling pathways involved in PACAP stimulation of PRL promoter activity in αT3-1 Cells via PAC1 receptor. In αT3-1 cells, PACAP stimulate PRL promoter activity through PAC1 receptor. PACAP induction of grass carp promoter activity was mediated by AC/cAMP/PKA pathway, with a crosstalk to the CaM/CaM K-II cascade via VSCC activation and [Ca^2+^]e entry. Intracellular Ca^2+^ release followed by PLC/IP3 activation also mediates the regulation of CaM/CaM K-II on PRL gene transcription. Moreover, PI3K/Akt and p70S6K signaling was involved in the post-receptor signaling of PACAP induction of PRL gene transcription. These signaling cascades triggered by PACAP stimulation could induce PRL promoter activation via the transcription factor CREB.

In summary, we have cloned the full gene of grass carp PRL and characterized its genomic organization and exon/intron junctions. The 5′ promoter obtained was used to set up a luciferase reporter system in αT3-1 cells to investigate the signaling mechanisms involved in PACAP stimulation of PRL gene transcription in grass carp. In our working model (Fig. 10), we have shown that PACAP induction of grass carp promoter activity was mediated by the CaM/CaM K-II cascade via VSCC activation and [Ca^2+^]e entry triggered by the AC/cAMP/PKA pathway. The CaM/CaM K-II pathway responsible for PRL gene transcription could also be activated by PACAP stimulation of the PLC/IP3 cascades, which leads to Ca^2+^ release from intracellular Ca^2+^ stores. In this study, we have also provided evidence that the PI3K/p70S6K pathway, but not MAPK or Akt, was also involved in the post-receptor signaling of PACAP induction of PRL gene transcription. Probably, the signaling cascades triggered by PACAP stimulation could induce PRL promoter activation via transactivation of the transcription factor CREB. Although the PACAP responsive sequence in grass carp gene has been mapped to the proximal region downstream of position −306 of PRL promoter using 5′ deletion, the cis-acting elements responsible for PACAP stimulation have been yet to be identified. Further investigations are clearly warranted to delineate the functional elements in PRL promoter conferring PACAP induction of PRL gene expression in grass carp.

## Materials and Methods

### Animals

One-year-old (1+) Chinese grass carps (*Ctenopharyngodon idellus*) with body weight ranging from 1.5 to 2.0 kg were acquired from local markets and maintained in well-aerated 200-liter aquaria under 12 L:12D photoperiod at 18 ± 2 °C. Since the grass carp at this stage was “prepubertal” (gonadosomatic index ≤ 0.2%) and sexual dimorphism was not apparent, fish of mixed sexes were used for blood sampling and pituitary collection. During the process, the fish were anesthetized in 0.05% MS222 (Sigma, St. Louis, MO) for blood sampling and sacrificed by spinosectomy for pituitary harvest according to the protocol (CULATR No.3890) approved for this study by the Committee for Animal Use in Teaching and Research at the University of Hong Kong (Hong Kong).

### Reagents and Test Substances

DMEM, fetal bovine serum (FBS), antibiotic-antimycotic, OPTI-MEM, and lipofectamine^TM^ were acquired from Invitrogen (Carlsbad, CA). Ovine PACAP_38_ was purchased from Phoenix Pharmaceuticals (Belmont, CA) and dissolved in double-distilled deionized water and stored frozen as 0.1 mM stock solution in small aliquots at −80 °C. Forskolin, H89, MDL12330A, 8-(4-chlorophenylthio)-cAMP (CPT-cAMP), A23187, Bay K8644, nifedipine, verapamil, 2-aminoethoxydiphenyl borate (2-APB), U73122, KN62, KN93, calmidazolium, TPA, GF109203, U0126, PD169316, SP600125, Ly294002, rapamycin and HIMOC were obtained from Calbiochem (San Diego, CA). The stock solutions of these pharmacological agents were prepared in a similar manner as that for ovine PACAP_38_ except that they were dissolved directly in dimethyl sulfoxide (DMSO). Stock solutions of test substances were diluted with prewarmed (28 °C) culture medium to appropriate concentrations 15 min prior to drug treatment. The final dilutions of DMSO were always less than 0.1% and had no effects on PRL promoter activity expressed in αT3-1 cells. Antibodies against total CREB (1:500; Calbiochem), phosphorylated CREB (1:500; Upstate, Milford, MA), and β-actin (1:15000; Oncogen, Cambridge, MA) were used for Western blot analysis. SiRNA for CREB were purchased from Dharmacon (Lafayette, CO, USA). The vector pRC/RSV.kCREB expressing a DN mutant of CREB, namely kCREB, was obtained from the laboratory of Dr. R.H. Goodman (Vollum Institute, Oregon Health Sciences University, USA). Other chemicals or materials used in this study were acquired from commercial sources with the highest quality available.

### Molecular Cloning of Grass Carp Prolactin Gene

Genomic DNA was isolated from the whole blood of grass carp using proteinase K digestion followed by repeated cycles of phenol:chloroform extraction. After isopropanol precipitation and desalting with 70% ethanol, genomic DNA prepared was used in intron trapping by PCR with specific primers designed based on the nucleotide sequence of grass carp PRL cDNA (GenBank No. EU074210). For the cloning of the 5′ promoter of grass carp PRL gene, genome walking was performed using a Universal GenomeWalker^TM^ Kit (Clontech, Palo alto, CA). Briefly, genomic DNA prepared was subjected to 15–18 hr digestion with EcoR V, Dra I, Pvu II, and Ssp I, respectively. After phenol:chloroform extraction, the digested DNA was ligated with GenomeWalker adaptor (Clontech) and used for nested PCR with GW1 (5′-GTAATACGACTCACTATAGGGC-3′) and GW2 (5′-ACTATAGGGCACGCGTGGT-3′) primers (for the adapter) and gene-specific primers for grass carp PRL, including GP1 (5′-GAGGGAGTGAAGTTTGTCTGA-3′) and GP2 (5′-GAGGGAGTGAAGTTTGTCTGA-3′). The PCR reactions were conducted according to the instructions of the manufacturer. After that, the PCR products were gel purified and subcloned into the pGEM^®^-T Easy vector for DNA sequencing. Based on the DNA sequences obtained from intron trapping and genome walking, the nucleotide sequence of the full-length gene of grass carp prolactin was compiled using MacDNasis Pro (Hitachi, Tokyo, Japan) and MacVector computer programs (Accerlrys Inc., San Diego, CA).

### Mapping of Transcription Initiation Site

Primer scanning and primer extension were conducted to delineate the transcription initiation site of the grass carp PRL gene. For primer scanning, total RNA was purified from the carp pituitary using TRIzol (Invitrogen) and reversely transcribed into first strand cDNA with Superscript III (Invitrogen). The RT samples were then analyzed by PCR using a 3′ anchor primer PS1 (5′-AAGTTTGTCTGAAAGTTGAGAGGGCT-3′) located in the downstream region of PRL 5′UTR and various 5′ primers located at upstream region of the PRL promoter (at position −720, −470, −210, and −90). During the process, parallel PCR with genomic DNA as the template was used as a positive control. In the same experiment, PCR with PS1 and P0, a 5′ primer located at the 5′ end of prolactin 5′UTR, was also performed to serve as a quality control for the RT samples prepared. For primer extension, total RNA was prepared from the carp pituitary. Using a plasmid DNA containing the PRL gene fragment covering the junction between the 5′ promoter and 5′UTR as a template, sequencing ladders (including C, T, A, and G) were constructed using PE1 (5′-CTGCAAAGTATAGTCTAGATCCT-3′) as a primer for cycle sequencing with a ^35^S Sequi Therm EXCELTM II DNA Sequencing Kit (EPICENTRE, Madison, Wisconsin). The samples obtained after primer extension were resolved in parallel with the sequencing ladders in 8% gel with 8 M urea by PAGE at 96 W. The gel was then vacuum-dried at 80 °C and autoradiography was performed with Biomax MR films (Kodak) at −80 °C for 2–3 days.

### Measurement of Prolactin Promoter Activity

A 1156 bp fragment of the 5′ promoter of grass carp prolactin gene was subcloned into the firefly luciferase-expressing vector pGL3.Basic (Promega) to generate pPRL(−1156).Luc. The construct was then used for 5′ deletion of PRL promoter by PCR cloning to yield pPRL.Luc reporter constructs carrying decreasing lengths of grass carp PRL promoter from position −1156 to −57. The reporter constructs with PRL promoter were used for transfection studies in αT3-1 cells. αT3-1 cells were cultured in 24-well poly-D lysine pre-coated cluster plates at a seeding density of 0.1 × 10^6^ cells/well/0.5 ml for transfection experiments. After 15 hr incubation, transfection was performed at 37 °C for 6 hr in 50 μl OPTI-MEM with 402 ng plasmid DNA and 2.1 μl lipofectamine (Invitrogen). The plasmid DNA was composed of 200 ng pPRL.Luc, 2 ng pTK.Luc (Promega), 20 ng pEGFP-N1 (Clontech), and 50 ng Pit-I expression vector with pBssK (Invitrogen) as the carrier DNA. The renilla luciferase-expressing vector pTK.Luc was included to serve as an internal control and the GFP-expressing vector pEGFP-N1 was used to monitor the potential variations in transfection efficiency between separate experiments. For the experiments with CREB siRNA, transfection was conducted in a similar manner except that the carrier DNA was replaced by SmarkPool CREB siRNA to make up the total amount of siRNA to 40 nM/well. Parallel transfection with siRNA control alone at 40 nM/well was used as the control. After transfection, the cells were exposed to drug treatment for 24 hr and dissolved in 200 μl lysis buffer (Promega). The lysate obtained was then subjected to luciferase activity measurement using a Dual Luciferase Reporter Kit (Promega) with a LB96 Microplate Liminometer (EG&G, Gaithersburg, MD).

### Site-directed mutagenesis of PRL promoter

To test the functional roles of potential binding sites in PACAP promoter, site-directed mutagenesis to introduce loss-of-function mutations or truncations in respective sites identified in carp PRL promoter were constructed separately using pPRL(−306).Luc as the template using the KAPA HiFi™ HotStart PCR kit (Kapa Biosystems) with the site-directed primers. With the construct of [pPRL(−306).Luc] as the template, the putative C/EBPalpha from −226 to −217 and AP1 from −136 to −130 were mutated from CTTTGCATTTA to CTTTACCGTTA, and from TGATTAA to AAGGTAA, respectively to make the mutation constructs^51,52^. Further, the C/EBPalpha from −226 to −217 and AP1 from −136 to −130 were truncated from CTTTGCATTTA to CTTTA, and from TGATTAA to TAA, respectively. The mutation and truncation constructs were then subjected to transfection and dual-luciferase assay to evaluate the effect of oPACAP_38_, compared with the wild-type construct [pPRL(−306).Luc] and [pPRL(−166).Luc].

### Inositol Phosphate Measurement in αT3-1 Cells

Transfected αT3-1 cells were labeled with 2 μCi/well of myo-[^3^H] inositol (DuPont/NEN) in myo-inositol free DMEM medium containing 10% fetal bovine serum. After incubation for 12 hr at 37 °C, myo-[^3^H] inositol labeling medium was removed, and αT3-1 cells were washed two times with prewarmed phosphate buffered saline (PBS; pH 7.4) to remove trace of the excessive myo-[^3^H] inositol. The transfected cells were then incubated in DMEM containing 0.1% BSA and 10 mM LiCl (to inhibit inositol phosphate degradation) for 20 min at 37 °C before drug treatment. After that, the transfected cells were treated with ovine PACAP_38_, VIP, and GnRH for 45 min. Drug treatment was terminated by removing drug-containing medium followed by addition of 0.5 ml/well ice-cold perchloric acid solution (0.5 M HClO4, 5 mM EDTA, 1 mM diethylenetriamine pentaacetic acid, and 0.18 mg/ml phytic acid) and incubated on ice for at least 5 min. The samples were then neutralized with 0.5 M KOH and incubated for 20 min at 0 °C to ensure a complete precipitation of KClO_4_. After a brief sedimentation, the supernatant containing total inositol phosphates was applied onto Dowex anion-exchange columns (AG 1 × 8 resin; Bio-Rad). After sample loading, these columns were washed sequentially with a “high-salt” elution buffer (0.1 M formic acid and 1 M ammonium formate) into vials containing scintillation cocktail. Levels of radioactivity in these samples were measured using a liquid scintillation counter (Beckman).

### Western Blot of pCREB and tCREB Protein Expression in Primary Grass Carp Pituitary Cells

Grass carp pituitary cells were prepared by trypsin/DNase II digestion method and seeded at a density of 2.5 × 10^6^ cells/well in 24-well plates and incubated for 48 h with or without drug treatment^53^. The cells were lysed in RIPA buffer (50 mM Tris-HCl, 1% Nonidet-P40, 0.25% sodium deoxycholate, 1 mM EDTA and 150 mM NaCl) containing a cocktail of protease and phosphatase inhibitors (1 μg/ml aprotinin, 1 μg/ml leupeptin, 1 mM Na_3_VO_4_, 1 mM NaF and 1 mM PMSF). Same amount of protein in each well was subjected to 10% sodium dodecyl sulfate-polyacrylamide gel electrophoresis (SDS-PAGE) and transblotted onto an Invitrogen™ PVDF membrane by low-current eletrotransfer at 50 mA for 1 h. After blocking with 5% bovine serum albumin in PBS-Tween 20 buffer (PBST) for 1 h at room temperature, the membranes were incubated with the primary antibodies against pCREB and tCREB overnight at 4 °C. After three-time washing with PBST, the membranes were re-incubated with corresponding secondary antibodies for 1 h at room temperature and introduced for signal development^54^.

### Data Transformation and Statistical Analysis

For transfection experiments, luciferase activity was measured in terms of “arbitrary light unit”. The data for firefly luciferase activity were transformed as a ratio of renilla luciferase activity expressed in the same sample (referred to as “FF/RL Ratio”). The normalization was conducted to control for potential variations in transfection efficiency between wells. Since no significant differences could be noted in the expression levels of renilla luciferase activity, the data were simply transformed into a percentage of the mean value in the control group (as “% Ctrl”) for statistical analysis. Data presented (Mean ± SEM) were pooled results from four to six experiments and were routinely analyzed by Student’s *t* test or ANOVA followed by Fisher’s least significance difference (LSD) test. Differences were considered significant at p < 0.05.

## Electronic supplementary material

Supplemenary Information
