# Supplementary material for: Grass Carp Prolactin Gene: Structural Characterization and Signal Transduction for PACAP-induced Prolactin Promoter Activity
Source: Sci Rep. 2018 Mar 15;8:4655. doi: 10.1038/s41598-018-23092-0 (PMC5854708; doi:10.1038/s41598-018-23092-0)
Supplement: Supplementary file 1 — Supplemenary Information [file 41598_2018_23092_MOESM1_ESM.pdf]

Grass Carp Prolactin Gene: Structural Characterization and Signal Transduction for PACAP-induced Prolactin Promoter Activity

Chengyuan Lin [a,b,c # *], Jin Bai [a #], Mulan He [a], Anderson O. L. Wong [a*]

[a] School of Biological Sciences, University of Hong Kong, Hong Kong.

[b] Clinical Division, School of Chinese Medicine, Hong Kong Baptist University, Hong Kong.

[c] YMU-HKBU Joint Laboratory of Traditional Natural Medicine, Yunnan Minzu University, China.

P1-F

1 aagcagaaagcaaagattcaagcaagacgagaataaagaaaccagttaaaatgATGGCTGAAGGA

M A E G

66 TCTAGACTATACTTTGCAG*gt*aagcaaaccttatcttcctcagtctaacctttcccgccacatt

S R L Y F A

131 ttagactttatctctattaccttgttacatcccctttcggttcccccattttcagttcatgctta

196 cttgatttcagctccctctatctgcctttcttcttttaccatactcctaatgacattactgttta

P1-R

261 actgttccttttgtctttgcttttc*ag*TGACCGTCCTGATGTGTGCGTTTGTCTCAATCAACGG

V T V L M C A F V S I N G

326 TGTCGGTCTGAATGATTTACTGGAAAGAGCCTCTCAACTTTCAGACAAACTTCACTCCCTCAGCA P2-F

V G L N D L L E R A S Q L S D K L H S L S

391 CCTCTCTCACCAATGACCTG*gt*cagtactaaacaccctctaaaaaaaaaaatgtgtgagtgtatg

T S L T N D L

456 tgttgttacaataacgttgactatgatgatggaaagaacccaaagcaggtttattaaagtcagtt

521 taaaagtatgagaatgtatccagatcaaacaggtaagtaataatggcagagcagtatacagtcga

586 gtccttggatgatagtaacactcactatacatgaacacacgaggaaaggacaaacgaagagatga

651 caatgctggagtggaatggaacaggacgatggaccaggaacaggcaagtagtctttcaggttggt

716 attcacaggatgttgagtccgtaatcattgtttacagtgagtgtcttttatagggtattgatgag

781 actgtgataggatcatgcattgacagacctgagtagagtcggtgcgattgaggatgatggcagaa

846 gcggaaagagcccggtggagccagaggggtggtggatcgaggcacagcaggagatccacaagtcc

911 aaagcggtgccagagcaatgactgaccatggtggagcagagggagtgcggggccaaggtggagtc

P3-F

976 ggtgggtctgagggccgaggtgaagtccagggctcagaggcttgaggtgaaaccaggggatcctc

1041 atataaaaggtggagctgcaacttggaaatccaaggcgaatccgagcagcagggcagaaacacag

1106 gaggagtcaaggggctgaagggagacagccgaggtgaggcagaggagctggatggcttaggcaga

1171 ggagggagagggaagctgggtgggacaatcctcaatgaagaacacttggagctgggcagagccaa

1236 tggcaataaaggagacttgaggctgacagcggcaatgaaagagcttggaagctggatgggaccac

1301 tgggtcatacagacagctggaaggcaccagcagaggaggaggctcaaggacaggaacaaaggtgg

1366 gaactggctcagtgtcatccaggtcagtaagccactccagtcactccaactccccaatattccca

1431 cttggacgaatgtggggaccggcccactcacctggacagactcggctttctctgggtttgaggca

1496 agcattggctcaggctctgtagcaaaattgctgggtggaggcggctttgggattggaagaggggg

1561 cttggagatgactagaggagatactcgaactggggagatggactggtgggtctgctcctccacta

1626 tccccactgtgttggaagatccataaacccacagggtgtaattgatgaagtccacaaggaacta

1691 taaccctcattcaacagtaacatctggccgaggatctcatccatctcactccacaaagatgtact

1756 gtacttaagagcctcatcctagcacgactcataatagatctcaaggaaatcctccacatagtctt

1821 ccagagggcggccatattgggtgagggtgagcagggactcatcagtgacagagagtaaaatcagg

1886 gagtgaaaaatgttggtggtgcctgctgggttcacagtgttggtaggtctggccatctgtaacat

1951 tgacgcagacaatggatgatggaaagaacccaaagcaggtttattaaagacacaggtgagcatac

2016 aggtaagacaatggatccagatcaaacaggtaagtaataatggtaatggcaagcagagcaatata

2081 caatagagtctttggatgatagttacactcactatacagtagtgatattatgtatgacactgaag

P3-R

2146 ctttgaagcacgtattgagcgcgcagcacaatctccagtgaagacttggacgtgttagacttatt

2211 tgtgcacataacatgaatccatttatatagtgggtggctgcttcatccgacagaactttttctac

2276 aagcttttagctgctttatttttactaaccaccagatggccctgttttcaacactctagaagct

2341 ttgagtctgagctttggggaaagcctcagtgctttatgagcctttatttccccatccctactata

2406 gaggaacacacaaagagagggcaaatgagcagatgacaatgctggaattgaagacaatggcaaac

2471 aaggaaaataatatttcagcttggtatcctgtggtcataatgataaccagacaatgagttaaatg

2536 agtgaatgtcttttatatgttattgatgggactgtgatagaatccatgtgtgcttgatgaaagca

2601 ggtgagaatcgtgagggatgctaggtaatgtagtttgtgagtgagagtgcagcaagtgaggcaga

2666 gtccagtatgtgtgtgagagttgcatgcccactgggttatttttgacttcagttttatccaaata

P2-R

2731 tgtatttttttcatgaataagtttgtttgacattaacctctcaaccac*ag*GATTCTCACTTTCCT

D S H F P

P4-F

2796 CCTGTTGGGAGGGTAATGATGCCCCGTCCGTCGATGTGCCACACATCCTCCCTTCAAATTCCCAA

P V G R V M M P R P S M C H T S S L Q I P N

2861 TGACAAAGACCAAGCCCTGAAAGTGCCG*gt*aagaacacactttactgcatccatcttcctctttt

D K D Q A L K V P

2926 taaaatgtaaaaatactttctcctattccagctaaaataaatatatatatttttttaaattacaa

2991 atagctattagacattatgcagcataaataaaatatattttaatttaattgtaattactaaattt

3056 atactttaatttatataacatataaaacaaactatataacataaaacaaactatacaaaaacata

3121 ataaatataataaccataatttaaaaaagacatttatgtccctcctttgaaagtcacccaaaaac

3186 aggtctcttcaaaaagttcttaaagagatcataaaagaaatccatatgaatcaagtttaatctaa

3251 gtcttctgaagagacacaatcgcttcacatgatgaagagattttaatttaggattttattcacat

3316 ataaacattgatcaaagtgcatcactgagcacattaaccctctggtattattcatttggcagtag

3381 tttttttttgttcagtaaaatcaatgtaatatatttttgttcaataatacttttttattttgtatt

3446 ttgagagtatttaataattcatatttatacaattttgatcaataatgatgatgtttttactattg

3511 aaatttttttcctttccaattaaagcagtatttcattttaaaagaagagagacaatgcctttaaa

3576 atccaatgggtctatcttgcactcgatattttagagtgtcaccccagccttcactgctgtgtgct

3641 tttttccctaaatcgtcacaaaatgctctatattttgcctatttcccattaatttcctacaccg

3706 gtcattttgagcgaaacaacaagtgtgactatttctttacaaccattaagctttttcaaataaa

3771 gtccaccatttttttgtgtgtgtggtcacatagcgactgctgcaaaagttttcataccatcccaa

3836 tgaagtagcgattttaaaatttcaaaatgttttttttttttttttggtctaaaatgaccaaac

3901 aacaccagagggttaattatgttacatgtaggctcagctgtagactacttgcttgaacaaacctc

3966 actggttcttccagaaaatgcaaagtgacatgttatctaatttgtttctgc*ag*GAGGATGAGTTG

E D E L

4031 CTTTCTTTGGCTCGGTCTCTGCTCCTGGCGTGGTCCGATCCCCTCGCCCTCCTCTCCTCTGAGGC

L S L A R S L L L A W S D P L A L L S S E A

4096 GTCCAGCCTGGCACATCCAGAACGCAACACCATTAATAGCAAGACCAAAGAACTGCAAGAAAACA **P5-R**

S S L A H P E R N T I N S K T K E L Q E N

4161 TCAACAGCCTGGGTGCAGGTCTGGAGCATGTCGTTCACAAG*gt*gagtgcgcttttaggaacagct

I N S L G A G L E H V V H K

4226 ggaatgttgattgcaagagggcaatatgaagtaaatcctaatgattttggatataaacataaag

4291 aaatctaacgtacacctgtaagcagggtgtatttatacgttgtccctttctctcttctctac*ag*A

4356 TGGGCTCATCCTCAGACAACCTGTCCTCTCTCCCTTTTTACAGCAACAGCCTTGGCCAGGATAAA

M G S S S D N L S S L P F Y S N S L G Q D K

4421 ACCTCTCGACTTGTCAATTTCCATTTTCTGTTGTCCTGCTTCCGCAGGGACTCCCACAAAATTGA

T S R L V N F H F L L S C F R R D S H K I D

4486 CAGTTTCCTCAAAGTTCTGCGCTGCCGGGCAGCCAAGAAGAGACCTGAGATGTGCTAGAGTGAAA

***S F L K V L R C R A A K K R P E M C * *** **P4-R**

4551 ATGCTACTCTGCTTCTCTCATTGTGGATGTTAAGTTAAAATGGCAGAGCAGTGAGTGATTTGAAA

4616 TGTTTCTTTATAATACCGCATGGCAAAATATGCCCCTTATTGTTTCAAAGATGTAGATATTTGAT

4681 TCACTTTCTTATATATTTGAATAAATAACTGGACCAACGGGCCATATTACTCTGTCCCCAGAGGT

4746 AAAAGACAACAAAAAGTAAACAAAAGTATTTCAATTAAATTTTAAAATTGAAGGTCTGCATGCAG

4811 TCATACATGATGAATAAGTGCACAGAGTATATTAAAATGTTTGACCATGCTCAGTTTGATAAGAC

4876 ATTTGTATAGTAAGTATTTATTAAGAAACGATGTTGGATCTTTAACACGTTTATTTTAACATTAT

4941 CTTCTGAAATGCAATATTTAAAGCTTTGCACCAATGTCAATAAATTTCTATCAAGCAAATTTAAA

Supplementary Figure 1. Nucleotide sequence of the grass carp PRL gene (excluding 5’ promoter). Introns are indicated in lower cases, and exons are shown in capital letters. Amino acids deduced from the coding region are shown in italic single letter codon, and the stop codon is indicated by “ * ”. Nucleotide numbering is defined with respect to the transcription start site deduced by primer extension. The primers using for the cloning and sequencing were underlined and marked in red color. P(n)-F represent the forward primers, and P(n)-R represent the revere primers using the reverse and complementary sequence of the marked nucleotides.

-1188 TTTGAATAAACAGTCAATTAATGTTACTGGAAGAACATTTGTTCATAACTTGAACAAACCTTCTGAG

-1121 AACATTCCCTGTTAGCTGGGTTGACATTTTGCATTAAAAGGAGGGCAACTAGTAAAAGAGCTTGCTC
Pit I

-1054 TCAGCAAATATCCAACTGAATTTAGGGGTGTGTGTGTGTGTGTGCGTGTGTGCGTGTGTGTGGTT
Sp I

-987 CAGACATACCCTACGTTATGGGGACAAATTGTCCCCAAAAATATCTGAAATCATTGTCCTTGTAGGG
GR Pit I

-920 ACATTTTTTTTGTCCCCATGAGGAAAACAGCTTATAAATCAATCATACAATGTGATGTTTTTTGAAA
AP-2

-853 ATGCAGAAAGTTTTTGTAATGGGTAGGGTTAAGGGATGGAATATACAGTATAAAAAGCATTATGTCT
C/EBPbeta Sp 1 Pit I

-786 ATGGAATGTCCCCATAAAACATGAAAAAATTTGTGTGTGTGTGCATTTTTCTGAACGGATTGTACAC
NF-kappaB Pit 1

-719 AGGAGCAAGGGGGGTAACAGAAGAATTTCCCCACACAGACGTGGAAAAATGTCAGCCTCTTAGAGCA

-652 GACCACCAGAGATAAATCAGAACCTGAAGAAGGCATAGAAGGGAAAGTTTCATAACAATGGGTTAAC
GTAT 1

-585 CAGTGCATCAGAAATAATAAAAAAGAAACAAAAAGGGCATAAAAGCTCTCAGTCCACAGCAGGACAT
Hb AP-4

-518 GACAAATGAGTGTTTCCTTTATGGGGGAAGGGTCTGTCATGTGTAGACTTTCTTTAAATGGCACGTG
Sp 1 El2

-451 GCTGCAAGACTGTGTGAACTGGTACCTTCATAGTGGTGCAGTCTATGAAAATTGTTTCATTTTCCAT
AP 1 Pit I

-384 TTTTTCCTCCATTTAGATATCCTAAAGATCCTAAAGTAATCGTATCACACTAAACACACATTTTTTT
HNF-3B

-317 AAACACAAAAACACAGATAAACCAAATGCTCCTTTTCACTTTAAATAAGCTCTATAATCAGAAGTTT
GTAT 1 TBP HSF

-250 CTCCATCCAGTAATTGAAAGAGTGCTTTGCATTTATTTATTTGGCACTTCATGTGACTGATGGTGAA
Oct 1 C/EBPalpha Pit 1 Hb

-183 TTTTGCCCCCACTGTGTCCTGTATCTACTCAAACAGCTTATCAATATTGATTAACCAAACTCTCAAA
HNF-4alpha1 GTAT 1 AP1

-116 ACAATGTTTTTCATTAGTTTCAGGATTGAACTAGTTTCAATACACCTGGAGTGTAAGACTTACTGCA
Pit 1 C/EBPalpha

-49 TATCCAAATGAAAGAAGTAGGGCTATATAATGAAAAGCTCAAGAGAGGA | aagca......
+1

Supplementary Figure 2. Nucleotide sequence of the 5' promoter region of grass carp PRL gene. Nucleotide numbering was defined with respect to the transcription start site deduced by primer extension. In the 5' promoter region, the putative cis-acting elements predicted with TESS site search (http://www.cbil.upenn.edu/tess/) are underlined. The possible TATA box is indicated by red letters. The 5' region of 5' UTR of grass carp gene (start with "+1") is shown in lower cases for reference.

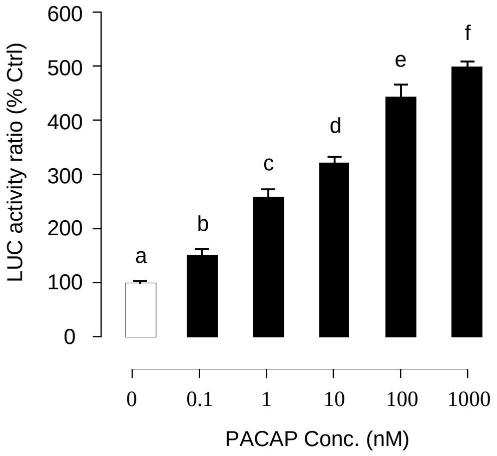

Supplementary Figure 3. Effect of grass carp PACAP38 on PRL promoter activity in αT3-1 Cells. αT3-1 Cells were transiently transfected with pPRL(-1156).LUC for 6 h by using lipofectamine. After 18 h recovery, the cells were incubated with grass carp PACAP38 dose-dependently. After drug treatment, cell lysate was prepared for dual-luciferase measurement Data are presented as percentage of control by conversing the ratio of firefly and renilla luciferase in the same sample. The results are expressed as mean ± SEM (n = 4) and different letters denote a significant difference at $p < 0.05$ (ANOVA followed by Fisher's LSD Test).

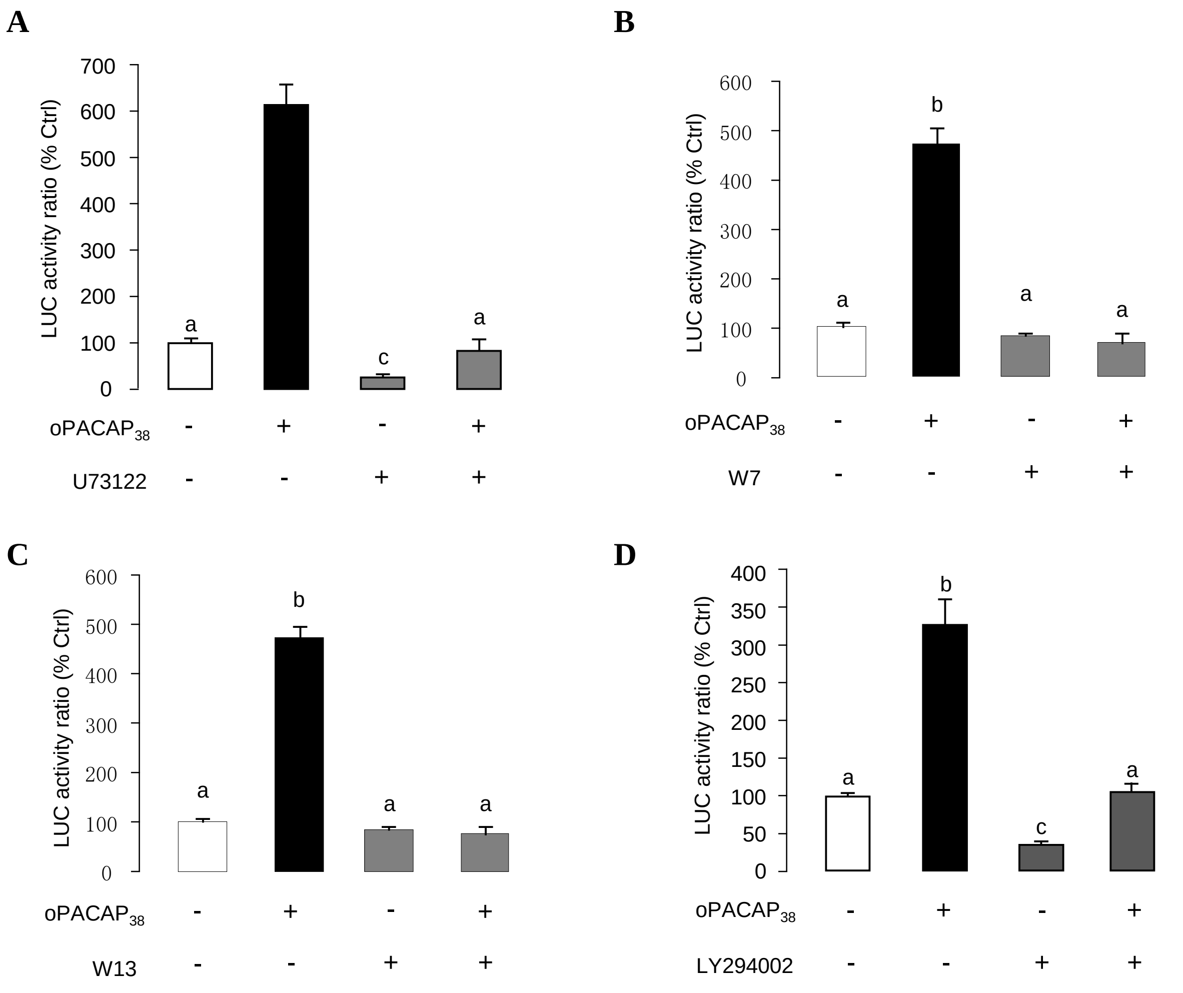

Supplementary Figure 4. Effect of U73122, LY294002, W7 and W13 on PACAP-induced PRL promoter activity in αT3-1 Cells. αT3-1 Cells were transiently transfected with pPRL(-1156).LUC for 6 h by using lipofectamine. After 18 h recovery, the cells were pre-treated with respective inhibitors for 15mins before co-incubation with oPACAP38 for 24 hours. After drug treatment, cell lysate was prepared for dual-luciferase measurement. (A) Effects of the PLC inhibitor U73122 (10 μM) on PACAP (100 nM) stimulated PRL promoter activity. (B&C) Effects of the CaM inhibitor W7 (30 μM) and W13 (100 μM) on PACAP (100 nM) stimulated PRL promoter activity. (D) Effects of the PI3K inhibitor LY294002 (10 μM) on PACAP (100 nM) stimulated PRL promoter activity. Data are presented as percentage of control by conversing the ratio of firefly and renilla luciferase in the same sample. The results are expressed as mean ± SEM (n = 4) and different letters denote a significant difference at $p < 0.05$ (ANOVA followed by Fisher's LSD Test).
